# Mechanism of Direct Acoustic Construction and Gradient Properties Tuning

**DOI:** 10.1002/advs.78376

**Published:** 2026-10-20

**Authors:** Dandan Wu, Lijuan Zheng, Guang Yao, Zijun Liu, Liyan Luo, Cile Liang, Chengyong Wang

**Affiliations:** ^1^ School of Electromechanical Engineering Guangdong University of Technology Guangzhou China; ^2^ State Key Laboratory For High Performance Tools Guangdong University of Technology Guangzhou Guangdong China; ^3^ Guangdong Provincial Key Laboratory of Minimally Invasive Surgical Instruments and Manufacturing Technology Guangdong University of Technology Guangzhou Guangdong China; ^4^ Smart Medical Innovation Technology Center Guangdong University of Technology Guangzhou Guangdong China

**Keywords:** acoustic‐induced curing and tuning mechanisms, circumferential fingerprint structure, directed acoustic construction, flexible sensing, gradient properties

## Abstract

Despite the effectiveness of microstructured elastomers for force sensing, current fabrication methods struggle to precisely control the gradient properties of complex microstructures due to limited energy‐field flexibility, resulting in uniform performance that hinders high‐sensitivity sensing. Here, directed acoustic construction (DAC) technology is employed to fabricate fingerprint‐inspired elastomeric microstructures with intra‐layer gradient properties, and the underlying mechanisms of acoustic curing, particle reinforcement, and property tuning are revealed. The acoustic‐induced curing of polydimethylsiloxane (PDMS) arises from synergistic physical, chemical, and mechanical effects. The incorporation of diamond particles into the PDMS matrix reinforces all three acoustic effects, thereby promoting polymerization and curing, which in turn broadens the tunable range of properties via acoustic waves. DAC enables omnidirectional patterning of microstructures and wide‐range gradient modulation, with feature sizes of approximately 200 µm. The resulting microstructure exhibits distinct inner–outer mechanical responses, producing a linear contact area–pressure relationship that enhances sensitivity and linearity. Compared with those of the uniform microstructures, the force sensing sensitivity is improved by 52.06%. When integrated onto a robotic fingertip as a functional sensing layer, it can distinguish fine textures and detect multidirectional forces. DAC enables site‐specific quantitative modulation and rapid fabrication (within 10 min), expanding its applications in micro/nanofabrication and flexible sensing.

## Graphical abstract

Directed acoustic construction enables omnidirectional gradient tuning of fingerprint‐inspired microstructures. By investigating the DAC mechanisms, diamond particles are found to enhance acoustic physical, mechanical, and chemical effects, promoting acoustic curing and broadening the range of gradient property tuning. The resulting gradient deformation improves force‐response linearity and sensitivity, advancing tactile sensors for robotic applications.

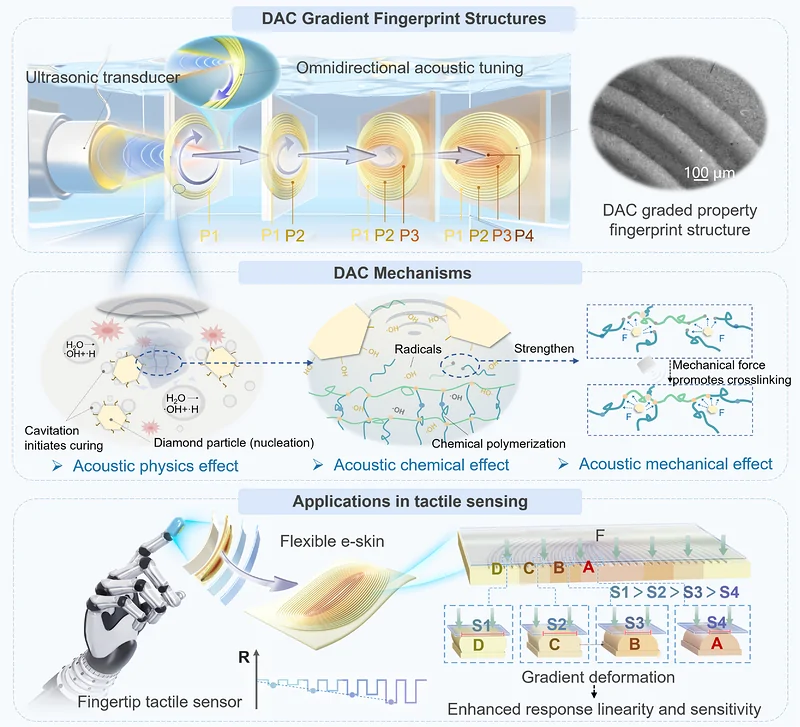

## 1 Introduction

The inherent flexibility, deformability, and stability of flexible thermosetting materials position them as ideal candidates for advanced applications, including wearable flexible sensors [1, 2] and intelligent robotics [3, 4]. Inspired by biological structures such as human fingerprints [5], creating microstructures on elastomer surfaces increases the contact area between the sensor and the target object, and furthermore, the arrayed microstructures increase the density of information sensing [6, 7]. The introduction of microstructures has been demonstrated to significantly increase the sensitivity and linearity of force sensing [8, 9]. Here, sensitivity refers to the sensor's responsiveness to changes in the input signal, typically manifested as the ratio of output change to input change. Linearity is reflected in the output more closely approximating an ideal linear relationship with the input.

However, conventional methods for fabricating microstructures, such as thermal curing [10], elastomer casting [11], and additive manufacturing [12], are restricted by a uniform energy distribution (heat, force, or light) within the material, inevitably resulting in structures with uniform properties. In contrast to such uniform structures, most microstructures in nature exhibit progressive, gradient properties, which are key to their remarkable functionality [13, 14]. Rational structural design and fabrication strategies have become essential for achieving advanced sensing functionalities [15]. Currently, some studies have achieved progressive compression of microstructures through multi‐scale structural design or gradient material composition, resulting in a gradient increase in contact area that manifests as a linear increase in electrical signals, thereby attaining high linearity and sensitivity across a low‐pressure range [16, 17, 18]. However, conventional fabrication strategies such as layer‐by‐layer casting or multi‐template stepwise thermal curing rely on complex mold design [19], multi‐step material synthesis [20], or multi‐step assembly processes [21]. These methods involve complex procedures and lack real‐time control and design flexibility during fabrication.

Despite the enhanced controllability of 3D printing techniques, the uniform energy deposition inherent to each printed layer makes it difficult to achieve flexible intra‐layer property tuning in thermosetting materials [22, 23]. Unlike energy sources such as light and heat, printing methods utilizing ultrasound as a direct energy source have garnered widespread attention in recent years. These techniques leverage the high focusing capability, flexibility, and penetrability of ultrasound, opening new avenues for the flexible fabrication of micro/nanostructures. Representative examples include direct sound printing (DSP) [24], holographic direct sound printing (HDSP) [25], and deep‐penetrating acoustic volumetric printing (DVAP) [26]. Habibi et al. [24] proposed the DSP technique, which achieves rapid printing of thermosetting polymer structures (e.g., PDMS) by harnessing the transient extreme energy generated within a microscopic cavitation zone using focused ultrasound. However, the DSP technique is limited to a single acoustic focal region, resulting in constrained manufacturing efficiency and precision for complex structural components. To overcome this limitation, Habibi et al. [25] further proposed the HDSP technique, which patterns the acoustic field using acoustic holograms. By programming the acoustic holograms, the required acoustic field for various geometric shapes can be actively synthesized in real time, enabling the construction of more complex shapes. Currently, DSP and HDSP focus primarily on the fabrication and validation of geometries using resin materials such as PDMS and epoxy resins, with little attention given to the tuning of gradient properties in microstructures or the underlying mechanisms. DVAP [26], on the other hand, is applied mainly to specialized materials such as self‐reinforcing acoustic inks to rapidly construct structures with specific biomedical functions inside biological tissues. The active regulation of material properties, however, has not been a central focus of this approach.

Recently, our team proposed a novel strategy for rapid fabrication using ultrasound—an original technology known as “Sound Continuous Production (SCP)” [27]. Unlike DSP, which relies on voxel‐by‐voxel scanning within a single acoustic focal region, or HDSP, which achieves regional polymerization through acoustic holograms, SCP utilizes the unique advantages of sound waves—non‐contact transmission and high focusability—to achieve 360° omnidirectional, multi‐scale, and multi‐material continuous forming. The difference between SCP and existing techniques lies in the fact that SCP operates with full degrees of freedom in acoustic wave manipulation, whereas DSP, HDSP, and similar methods are based on layer‐by‐layer or voxel‐by‐voxel fabrication. Moreover, DSP, HDSP, and DAVP primarily focus on structural fabrication, whereas SCP additionally enables regulation of material properties. Therefore, these features of SCP offer a more flexible and tunable approach for microstructure manufacturing. However, when aiming to construct microstructures with gradient properties, two critical issues need to be addressed: how to achieve site‐specific and quantitative property regulation via acoustic waves and what the underlying mechanisms of acoustic‐induced curing behavior and property tuning are.

In our previous studies, the SCP technique was successfully employed to fabricate various structural samples of PDMS, epoxy resins, and acrylates. We observed three distinct effects—acoustic physical, acoustic chemical, and acoustic mechanical effects—during the direct ultrasonic curing process. In addition, it was found that acoustic waves induce molecular entanglements at the interfaces of liquid thermosetting materials, resulting in a continuous interface. Here, we focus on the mechanism of continuous interface formation during the SCP process; however, the underlying curing mechanisms of acoustic‐induced curing and property tuning are not completely clear.

Specifically, the synergistic interplay of acoustic physical, chemical, and mechanical effects in acoustic‐induced curing has yet to be systematically investigated. For example, how physical effects such as cavitation and bubbles caused by acoustic waves concretely promote material curing is unclear. Although sonoluminescence has been confirmed to occur during curing, the specific types of free radicals generated and the mechanisms by which they initiate chemical polymerization remain to be elucidated. Furthermore, while finite element simulations have previously demonstrated the occurrence of mechanical vibration effects induced by ultrasound [27], the potential role of acoustic mechanical effects in the polymerization and curing process requires further investigation. In addition, the introduction of diamond particles into the PDMS matrix has preliminarily confirmed their enhancing effect on the three acoustic effects [28]. However, the influence of such introduced secondary phases on acoustic cavitation behavior and evolution (formation, growth, and collapse), radical‐initiated polymerization, and acoustic mechanical vibration remains unclear. Therefore, the mechanism by which the particles achieve gradient regulation still requires further investigation.

Based on this understanding, this study employs a “Directed Acoustic Construction (DAC)” strategy, incorporating diamond as a reinforcing phase in the PDMS matrix to tune performance, thereby enabling complete freedom to fabricate ridge fingerprints with gradient properties. In the process of directed acoustic construction, acoustic‐induced PDMS curing arises from synergistic effects: acoustic physical effects (curing triggered by cavitation), acoustic chemical effects (free radical‐promoted internal polymerization), and acoustic mechanical effects (cross‐linking reinforced by acoustic mechanical vibration). The incorporation of diamond particles further amplifies these three types of acoustic effects and broadens the tunable range of internal molecular polymerization and cross‐linking networks via acoustic waves, thereby expanding the regulation window of gradient properties. The unique concentric gradient fingerprint configuration produces differentiated mechanical responses from the center outward, enabling a linear relationship between the contact area and pressure. Consequently, when integrated into a robotic fingertip for sensing applications, it exhibits higher sensitivity, enhanced linearity, rapid dynamic/static response, and accurate multidirectional force recognition, achieving precise roughness and fine texture recognition.

## 2 Results and Discussion

### 2.1 DAC Gradient Fingerprint Structures

Unlike the conventional additive manufacturing method of layer‐by‐layer printing, DAC features omnidirectional freedom and rapid fabrication, enabling one‐step fabrication of structures with continuous intra‐layer gradient properties. As shown in Figure 1a, an ultrasonic transducer on a six‐axis robotic arm moves along an elliptical path, directly acting on the liquid diamond/PDMS composite in the inner chamber. Under four ultrasonic energy gradients (P1–P4), the composite material on the fingerprint template undergoes sequential curing from the inner layer to the outer layer. After template removal, a diamond/PDMS composite exhibiting a ridge‐shaped fingerprint pattern with four‐level circumferential gradient properties is obtained. The feature size of the pattern is approximately 200 µm (Figure 1b). The diamond/PDMS composite samples before and after DAC are shown in Figure 1c,d, respectively.

**FIGURE 1:**
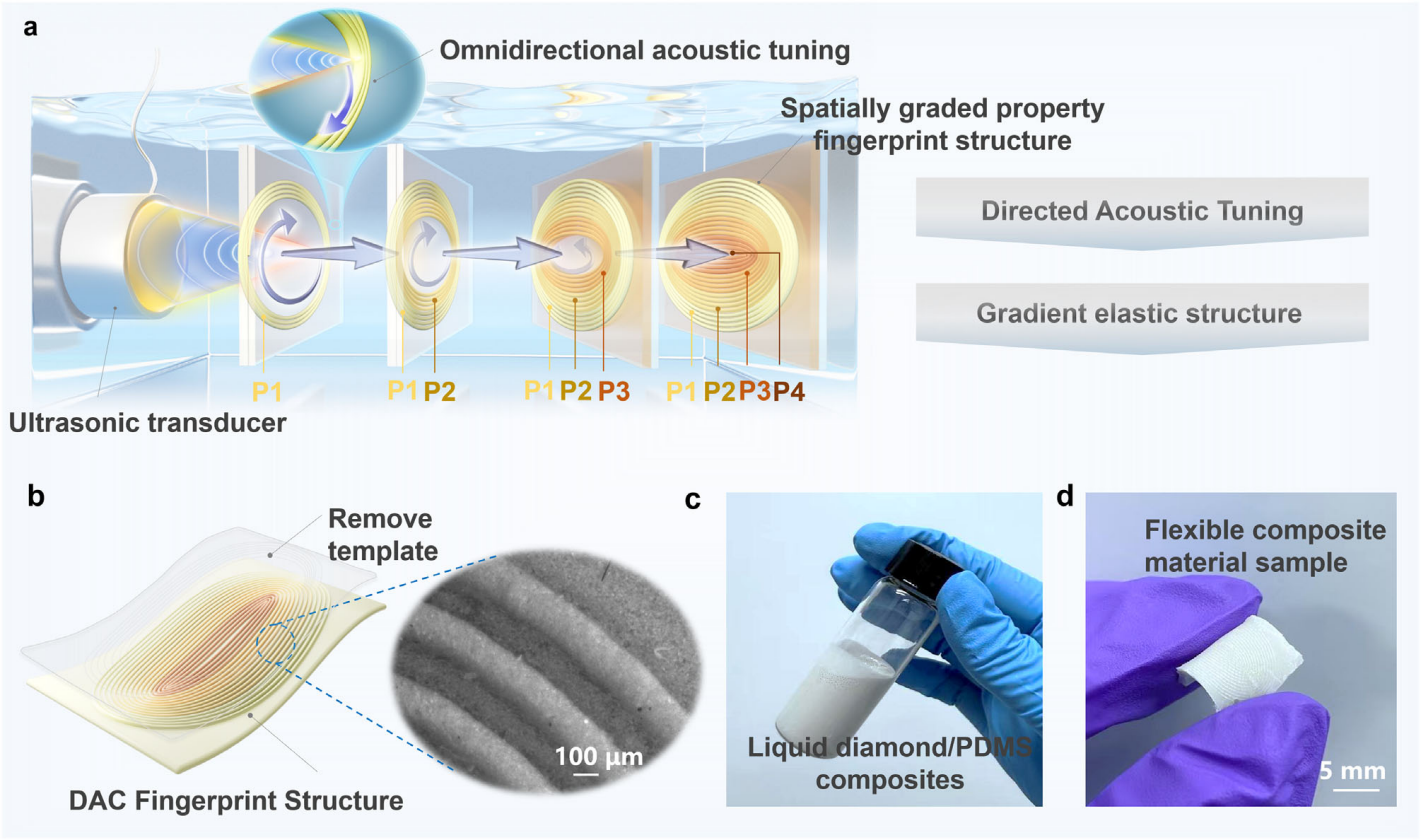
DAC ridge‐shaped fingerprint structure: (a) DAC process; (b) Surface morphology of fingerprint structure; (c) Liquid diamond/PDMS composite material (with 1 µm diamond particles); (d) DAC fingerprint structure sample.

### 2.2 Mechanisms of Acoustic Curing and Reinforcement in DAC

#### 2.2.1 Acoustic‐Induced Curing and Tuning Mechanism of PDMS

The continuous acoustic construction of ridge‐shaped fingerprint structures is achieved through stepwise point‐by‐point connections. To investigate the acoustic‐induced curing mechanism of diamond/PDMS composites at a single point, ultrasound was first focused on the liquid PDMS matrix material to exclude the influence of particles. We found that three types of acoustic effects exist during the curing process: physical, chemical, and mechanical effects (Figure 2a). Materials that respond sensitively to sound waves and exhibit such acoustically induced phenomena are herein defined as acoustic‐sensitive materials.

**FIGURE 2:**
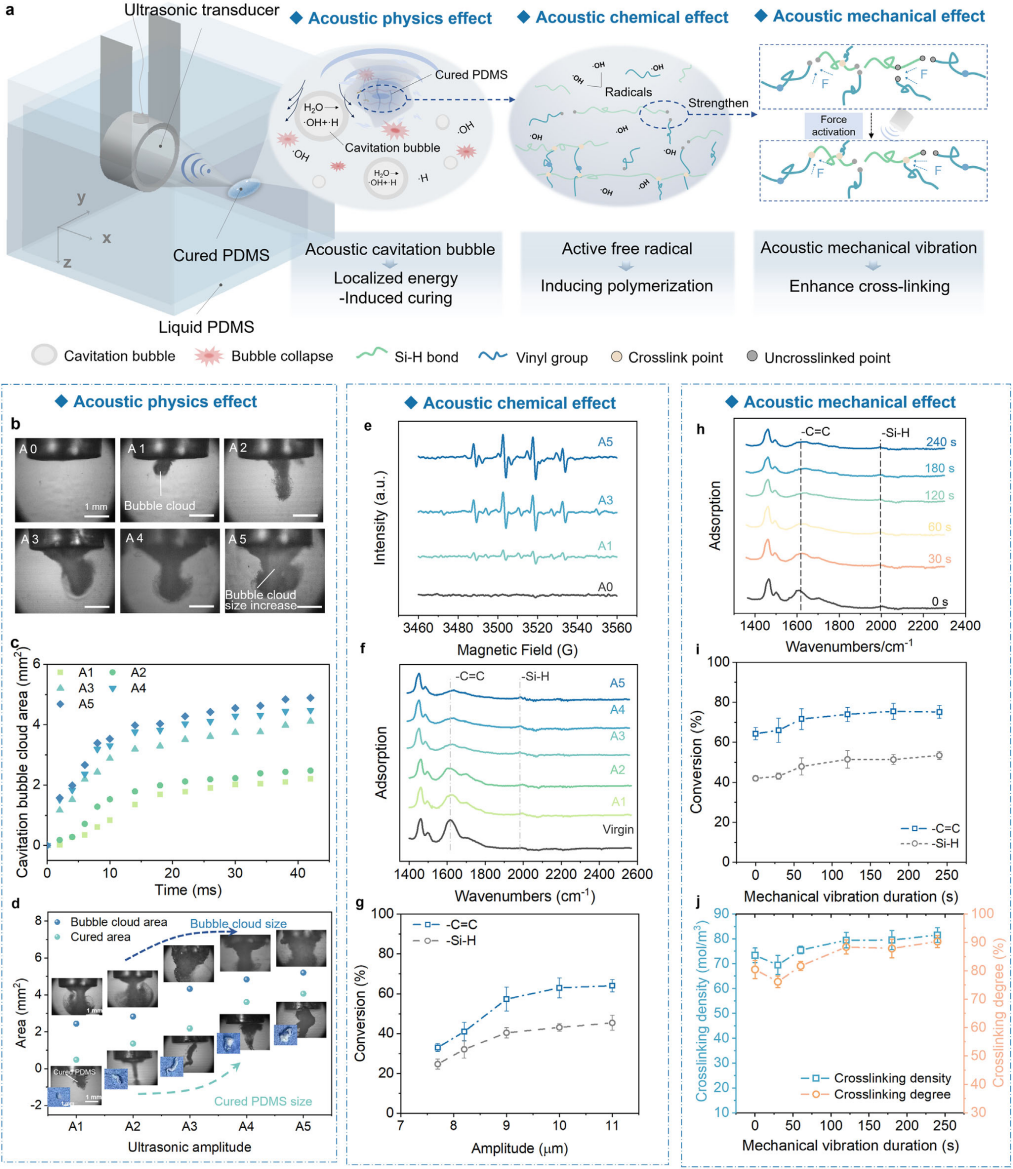
DAC mechanism of pure PDMS: (a) Illustration of acoustic physical, chemical, and mechanical effects triggered by ultrasound; (b) Evolution of bubble clouds in PDMS under different ultrasound amplitudes (A0–A5); (c) Time‐dependent variation in bubble cloud size; (d) Correlation between the bubble cloud area and cured PDMS size; (e) Radical signals in PDMS (120 s treatment); (f) FTIR spectra of PDMS under different ultrasound amplitudes; (g) conversion of reactive functional groups induced by free radicals; (h) FTIR spectra of the PDMS matrix under different durations of acoustic mechanical force; (i) Conversion rate of reactive functional groups under different durations of acoustic mechanical force; (j) Cross linking density and degree under different durations of acoustic mechanical force.

At the mesoscopic scale, the physical effect induced by sound waves manifests mainly as acoustic cavitation behavior in the material system (Figure 2a). When ultrasonic waves travel through a liquid medium, alternating positive and negative pressure cycles occur, generating dense and sparse wave regions [29, 30]. At sufficiently high ultrasonic intensities, these fluctuations lead to the formation of bubble clouds within the liquid, known as acoustic cavitation bubbles. The nucleation, growth, and collapse of cavitation bubbles generate transient high‐temperature (≈5000 K) and high‐pressure (≈10 000 atm) conditions [31, 32]. Here, the extreme physical conditions associated with acoustic cavitation are found to serve as localized hot spots, rapidly initiating the curing of thermosetting materials such as PDMS (Figure 2a). The distribution of cavitation bubble clouds in PDMS at 6 ms under different ultrasound amplitudes is shown in Figure 2b. With increasing amplitude from A0 to A5 (0–11 µm), the bubble clouds grow larger and more densely packed (see Figure S1 for details). The temporal variation in the bubble cloud size, which increases sharply within the first 0–10 ms before reaching a stable rate of growth, is shown in Figure 2c. Higher ultrasonic amplitudes intensify the stretching effect on liquid molecules during the rarefaction phase, promoting more nucleation events and the formation of larger bubble clouds. Accordingly, the localized energy generated instantaneously upon the collapse of larger cavitation bubble clouds directly induces rapid curing of the material, thereby forming a correspondingly larger cured PDMS volume (Figure 2d).

At the microscale, the underlying mechanism of this acoustic curing behavior lies in the effects of acoustics. The extreme thermodynamic conditions created by ultrasonic cavitation promote the generation of reactive free radicals such as hydroxyl (·OH) in the material, which in turn directly initiate the polymerization reaction (Figure 2a). Electron paramagnetic resonance (EPR) spectra of spin‐trapped radicals are shown in Figure 2e. Ultrasound triggers rapid free radical production, with a pronounced increase in the intensity of the hydroxyl radical signal. At an ultrasound amplitude of A5, the radical content is approximately 14 times that of the non‐ultrasonicated sample (A0). Hydroxyl radicals rapidly initiate reactions among active functional groups within the matrix, facilitating both thermally activated hydrosilylation between vinyl and Si─H bonds and the polymerization of vinyl groups. The FTIR spectra in Figure 2f show that the absorption peaks of the two reactive functional groups gradually weaken, indicating enhanced polymerization conversion (Figure 2g).

Additionally, the acoustic mechanical force generated by continuous ultrasonic vibration induces changes in specific molecular bonds, further promoting polymerization (Figure 2a). To eliminate the influence of acoustic chemical and physical effects in liquids, ultrasonic waves were directly applied to the cured material samples to determine the effect of acoustic mechanical vibration on material polymerization and cross‐linking networks. The cured sample A5, which exhibited the highest polymerization degree, was selected for further ultrasonic treatment. As shown in Figure 2h, the conversion rates of Si─H and vinyl groups increase with ultrasonic mechanical vibration duration, rising slightly within the first 30–120 s before reaching a plateau. However, the reinforcing effect of acoustic mechanical effects alone on PDMS polymerization and cross‐linking remains limited. The relative conversion rates of Si─H and vinyl groups increase by only 11.53% and 7.22%, respectively (Figure 2i), whereas the cross‐linking density and degree of cross‐linking increase by merely 10.19 mol/m^3^ and 11.1%, respectively (Figure 2j).

#### 2.2.2 Reinforcement of Acoustic Effects by Diamond Particles

Furthermore, the diamond particles were incorporated as a reinforcing phase into the PDMS matrix to investigate their influence on the three acoustic effects. Specifically, at the mesoscopic scale, the diamond particles were observed to intensify the acoustic cavitation behavior, accelerating the curing of the composite. This enhancement was evident in two ways. First, the incorporation of diamond particles creates additional nucleation sites for cavitation bubbles, thereby increasing the strength and uniformity of the cavitation distribution (Figure 3a). Diamond particles with sizes of 60, 120, and 180 µm were introduced into the matrix, labeled D60, D120, and D180, respectively, while a particle‐free sample was designated D0, all under the same ultrasonic amplitude of A1. High‐speed imaging, as shown in Figure 3b, reveals that at 42 ms, the addition of diamond particles produces a more intense and uniform cavitation effect than pure PDMS does (D0) (Figure S2). In particular, compared with pure D0, 60 µm diamond particles (D60) enlarged the bubble cloud size by 274.66% and expanded the cured region size by approximately tenfold (Figure 3c,d).

**FIGURE 3:**
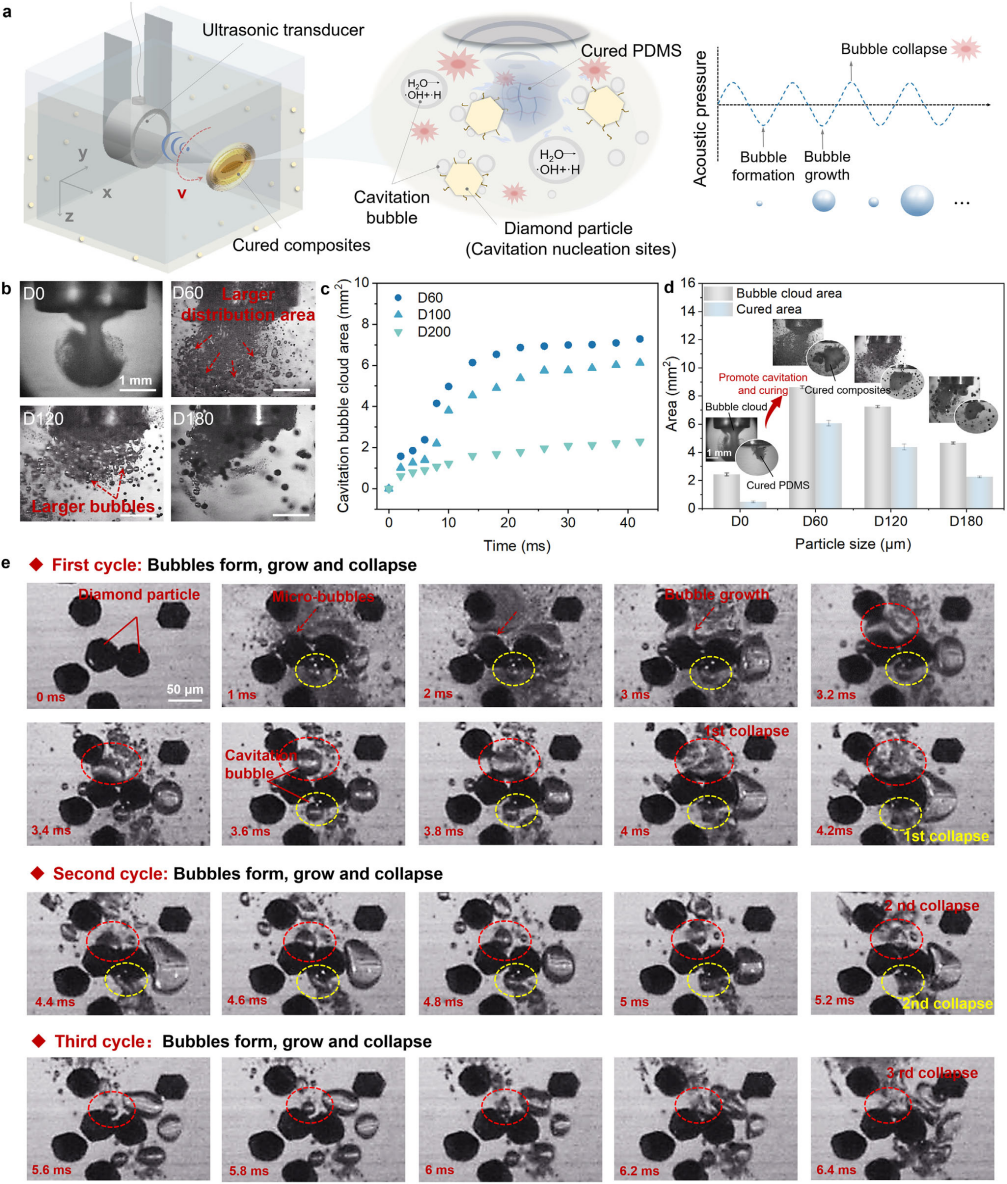
Enhancement of acoustic physical effects by diamond particles: (a) Illustration of cavitation behavior enhancement by the incorporation of diamond particles; (b) Variation in the number of cavitation bubble clouds in composite materials reinforced with diamond particles of different sizes; (c) Time‐dependent evolution of the number of acoustic cavitation bubble clouds in composites reinforced with varying particle sizes; (d) Correlation between the bubble cloud area and the cured diamond/PDMS composite size; (e) Evolution cycle of bubble formation, growth, and collapse on a single diamond particle surface.

The enhancement of cavitation by particles has been widely reported, yet these studies are predominantly focused on aqueous systems. In such systems, particles including SiO_2_, Au, and Al_2_O_3_ serve as nucleation sites to promote bubble formation, oscillation, and collapse, and the resultant shock waves can erode particle surfaces or facilitate dispersion [33, 34, 35]. Despite the different medium and purpose, a similar mechanism may underlie our observations. In this work, the enhanced cavitation uniformity and curing induced by diamond particles may also be attributed to the additional nucleation sites provided by their irregular surface features and chemical properties. To further verify this, 60 µm diamond particles were fixed at the bottom of the PDMS solution chamber to observe the formation of cavitation bubbles on the particle surfaces. At 3.5 ms of ultrasound exposure, tiny bubbles were observed to nucleate on the particle surfaces and progressively multiply with continued irradiation (Figure S3). This preliminarily confirms that the introduction of diamond particles provides more favorable conditions for cavitation bubble formation. Furthermore, at an equivalent volume fraction, smaller particles offer more nucleation sites, leading to higher bubble counts and density. This enhanced cavitation effect produces stronger shock waves and greater localized energy, thereby accelerating the acoustic curing process.

Second, high‐speed imaging further focused on localized diamond particles revealed that these particles induced more frequent nucleation, growth, and collapse cycles, a phenomenon not observed in the pure PDMS matrix. The evolution of cavitation bubbles on the surface of a single diamond particle captured by high‐speed imaging at an elevated frame rate is shown in Figure 3e. Taking the particle marked by the red circle as an example, three complete nucleation–growth–collapse cycles were recorded (Movie S1). During each cycle, the bubble diameter decreased progressively (45.5 µm → 23.5 µm → 11.5 µm), whereas the nucleation and collapse speeds increased, with corresponding periods of 4.2, 1, and 0.8 ms, respectively (Figure S4). In contrast, even with high‐impedance SiO_2_ particles, such multiple bubble oscillation cycles were not observed. This difference may be attributed to the extremely high acoustic impedance of diamond (about 4.7 times that of SiO_2_ [32, 36]), which induces stronger acoustic reflections at the particle surface (Figure S5). The resulting local pressure amplification promotes more facile and frequent cavitation nucleation. This multiple‐cavitation behavior leads to continuous energy release from the bubbles, effectively enhancing ultrasound‐induced curing of the composite.

At the microscale, unlike SiO_2_ particles, which mainly enhance physical cavitation but lack sufficient surface active groups to promote radical formation, diamond particles contribute both chemically and physically (Figure S6). From a chemical perspective, when diamond particles are added, intense ultrasonic shock waves or microjets break active surface functional groups, resulting in a higher concentration of hydroxyl radicals to promote polymerization (Figure 4a). To enhance this effect, diamond particles of smaller sizes (1–30 µm) were incorporated as the reinforcing phase, denoted as P/D1 to P/D30, while particle‐free pure PDMS was labeled P. Under the same ultrasound amplitude (A1) and treatment duration (120 s), the radical signals were stronger for the composites than for pure PDMS, with the peak intensity increasing as the particle size decreased (Figure 4b). Specifically, the radical content increases by a maximum of 174.51% compared with that of the pure PDMS sample (Figure 4c). This result is primarily attributed to the larger specific surface area of the smaller diamond particles, which increases the number of surface hydroxyl groups (Figure 4d). When subjected to ultrasound, intense shock waves or microjets cleave these groups, generating hydroxyl radicals. As shown in Figure 4e, at a fixed volume fraction, smaller particles yield a greater number of nucleation sites, thereby producing more radicals. Additionally, from a physical perspective, the intensified acoustic cavitation behavior resulting from diamond particle incorporation produces elevated localized energy, potentially leading to a higher concentration of free radicals. Consequently, the composites reinforced with 1 µm diamond particles (P/D1) achieve higher conversion rates of Si─H and vinyl groups during polymerization (Figure 4f) and offer a broader tuning range of polymerization via acoustic energy (Figure 4g).

**FIGURE 4:**
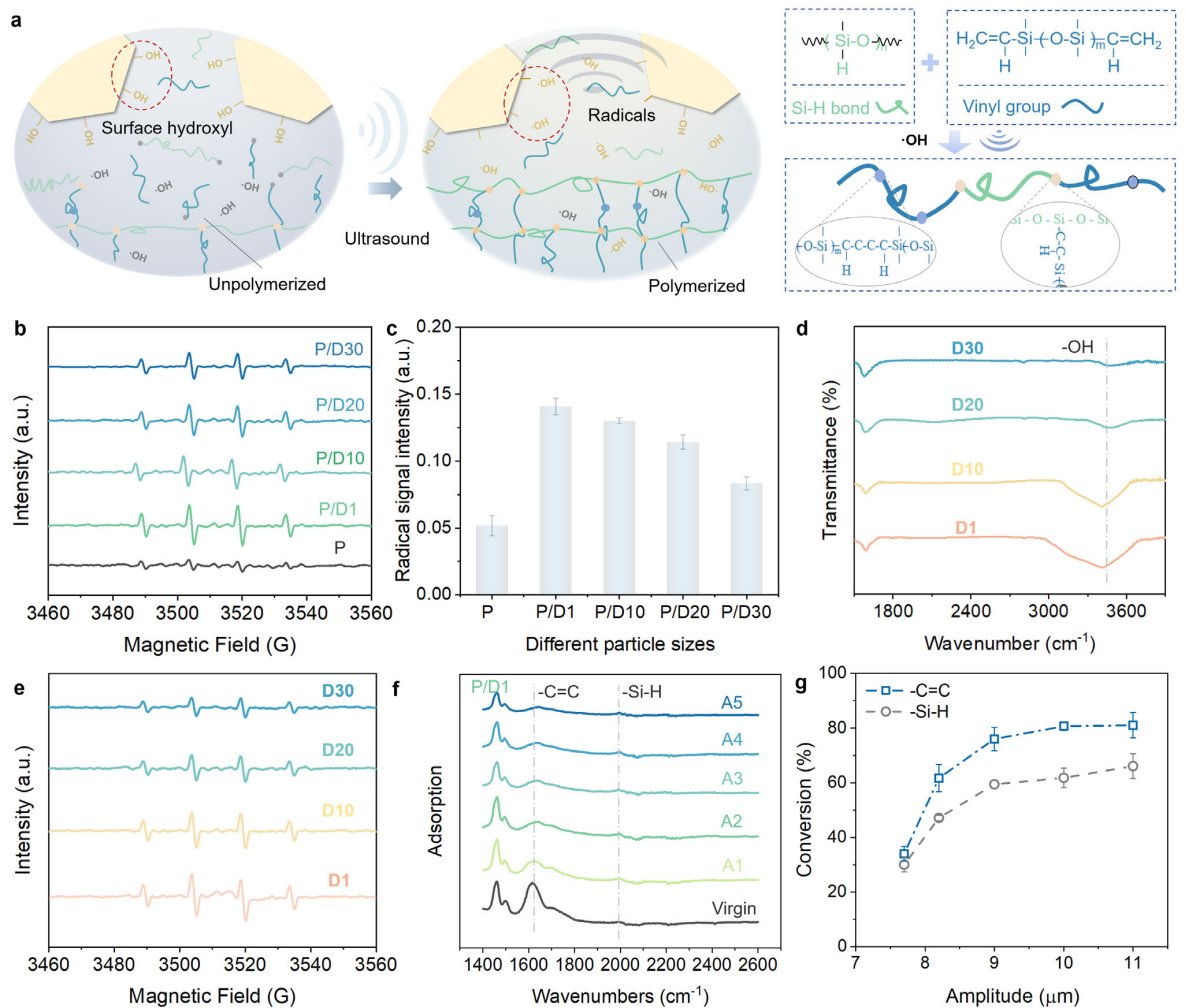
Enhancement of acoustic chemical effects by diamond particles: (a) Illustration of the enhancement of free radical production and polymerization by diamond particles; (b) Radical signals in composites reinforced with different particle sizes; (c) Corresponding comparison of radical content; (d) FTIR spectra of diamond particles; (e) Radical signals of diamond particles with different sizes (1–30 µm); (f) FTIR spectra of composites (P/D1 sample) under different ultrasonic amplitudes; (g) Effect of ultrasonic amplitude on the relative polymerization conversion rate in the composite.

Additionally, the incorporation of diamond particles also enhanced the acoustic mechanical effect within the material (Figure 5a). To evaluate this effect, the cured composites with 1–30 µm diamond particles (all fabricated under A5 amplitude) were subjected to ultrasonic mechanical vibration. FTIR analysis revealed that as the ultrasonic vibration duration increased from 0 to 240 s, the characteristic absorption peaks of reactive functional groups decreased significantly across cured composites with various particle sizes, indicating a further increase in conversion rate (Figure 5b–e). Notably, the reinforcing effect of the acoustic mechanical vibration on conversion is more pronounced in composites containing smaller diamond particles. Under continuous acoustic mechanical vibration for 240 s, the relative conversion rates of Si–H and vinyl groups in the composite with 1 µm diamond particles increased by 25.33% and 18.38%, respectively, nearly twice those of pure PDMS (Figure 5f,g). This improvement may be attributed to momentum transfer between the diamond particles and the acoustic field, which amplifies or redirects the acoustic mechanical forces. The enhanced mechanical effect promotes molecular chain motion and collisions (Figure 5a), resulting in diamond/PDMS composites with higher degrees of polymerization and denser cross‐linked networks (Figure S7).

**FIGURE 5:**
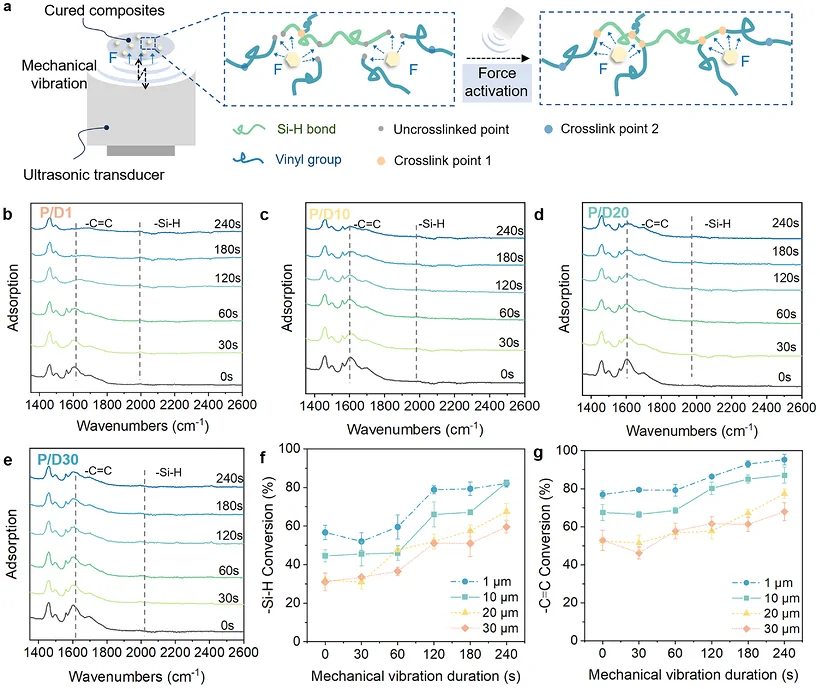
Enhancement of acoustic mechanical effects by diamond particles: (a) Illustration of diamond particles enhancing the effect of acoustic mechanical forces on molecular bonds; (b–e) FTIR spectra of composites under different durations of acoustic mechanical vibration (b) P/D1, (c) P/D10, (d) P/D20, and (e) P/D30; (f) Conversion rate of Si─H bonds; (g) Conversion rate of vinyl groups.

Overall, the acoustic‐induced curing behavior is thus a consequence of the synergistic interaction between the physical, chemical, and mechanical effects of the acoustics. At the mesoscale, acoustic cavitation achieves localized point curing through the transient high temperature and pressure generated by collapsing bubbles. At the microscale, active free radicals produced by acoustic chemical effects continuously drive polymerization and cross‐linking, forming the fundamental mechanism of acoustic curing. Moreover, ultrasonic vibration further strengthened the cross‐linked network in the cured region through acoustic mechanical effects. The introduction of diamond particles significantly enhances all three effects, expanding the tunable range of acoustic waves over microscopic polymerization and cross‐linking processes.

### 2.3 Acoustic Tuning of Gradient Properties in Fingerprint Structures

The incorporation of diamond enhances the regulatory range of the micro‐polymerization and cross‐linked network, thereby extending the tunable range of gradient properties induced by acoustic waves. The concept of a circumferential gradient ridge fingerprint structure formed through sound wave regulation is illustrated in Figure 6a. By precisely controlling the spatial distribution of the elastic modulus in local regions, the contact mechanical response of the functional layer can be effectively optimized. Compared with uniform microstructures with homogeneous properties, the circumferential fingerprint regions exhibit varying local stiffness under external pressure, resulting in a non‐uniform contact area distribution (S_1_>S_2_>S_3_>S_4_). This gradient feature allows the total contact area of the fingerprint structure to respond linearly to increasing pressure, providing a new design strategy for highly sensitive and rapid‐response sensing.

**FIGURE 6:**
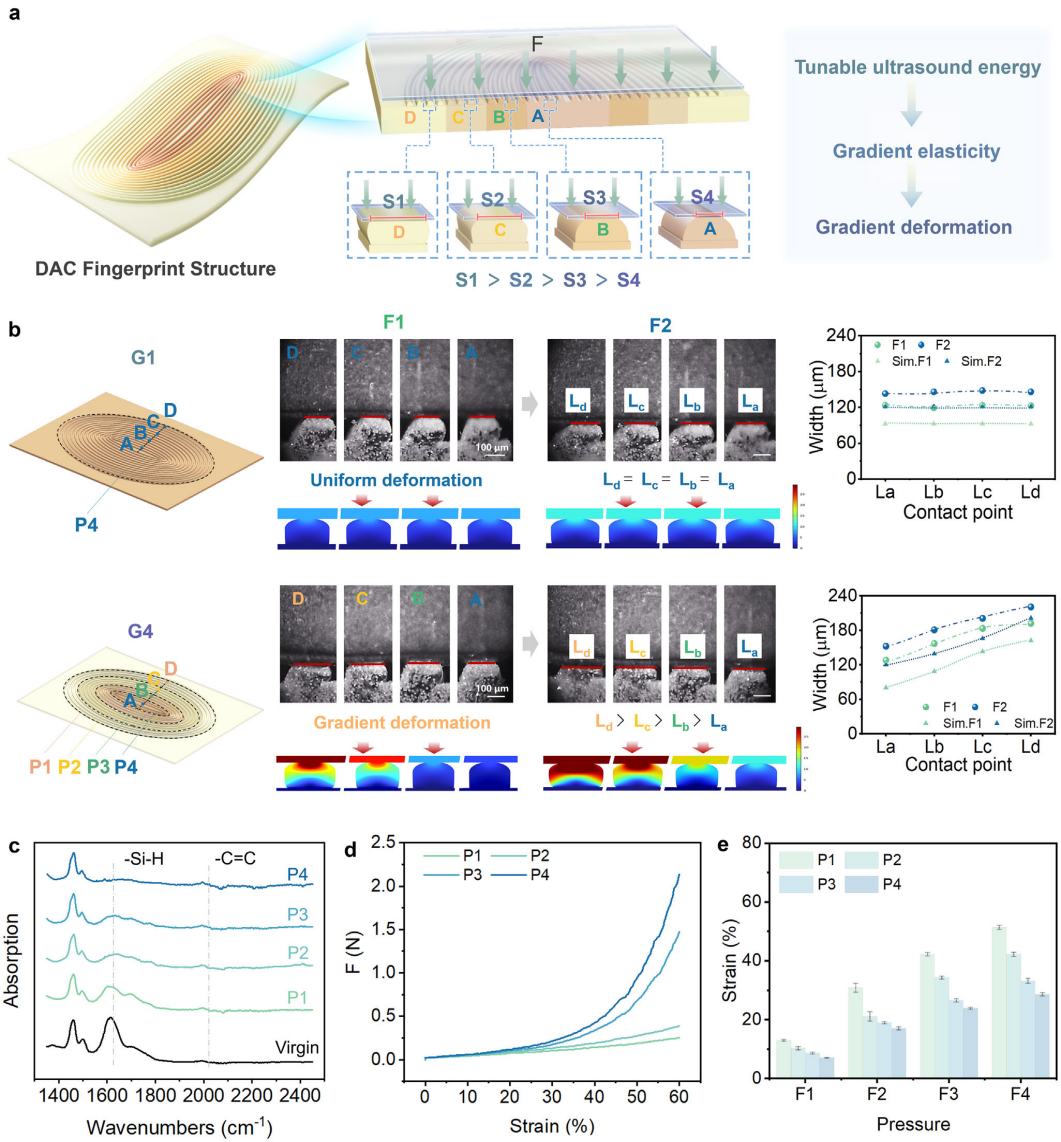
Gradient properties characterization of the fingerprint structural layer: (a) Illustration of the circumferential gradient elasticity; (b) Deformation evolution of the surface microstructure; (c) FTIR spectra of localized regions of G4 formed under different ultrasonic powers; (d) Force–strain curve; (e) Comparative analysis of the quantitative force–strain relationship.

To examine the gradient elastic behavior, high‐speed photography was employed to capture the deformation process of the structural layers with different elasticity gradients (G1–G4) (Figure S8). Here, 2 MHz high‐frequency focused ultrasound was employed to increase the fabrication precision of the microstructures. The ultrasonic power applied sequentially from the outer to inner regions was set as P1–P4 (5–17.5 W; see Figure S9 for parameter selection). As shown in Figure 6b, four evenly spaced ridge structures of identical dimensions were selected along the circumferential direction, with monitoring points (A–D) positioned radially every third ridge. Vertical pressure was then applied to record their deformation.

As depicted in Figure 6b, the deformation of the contact points exhibits a distinct gradient evolution with increasing microstructural gradation (Figure S10). For the homogeneous sample G1, when the pressure increases (F1: 0.05 N → F2: 0.1 N), all contact points (A–D) deform uniformly (Movie S2). In contrast, the graded structures exhibit differential deformation. The four‐level gradient sample G4 displays the most distinct gradient behavior under both F1 and F2. At F1, the contact lengths L_a_, L_b_, L_c_, and L_d_ are 128, 152, 183, and 198 µm, respectively—a 34.8% increase in total contact length compared with G1 (Movie S3). The finite element simulation of the G4 sample under compressive loading also revealed gradient deformation behavior (see Figure S11 for the finite element model). Specifically, the microstructures at different local positions, fabricated under different ultrasonic powers (P1–P4), showed a gradient increase in contact area, consistent with the observed experimental results (Figure S12). Details of the finite element model setup and boundary conditions are provided in the Supporting Information.

The local properties of the fingerprint structure across different regions correspond directly to its polymeric structure. As shown in Figure 6c, the absorption peaks of the two reactive functional groups (─Si─H and ─C═C) gradually weaken as the ultrasound power increases from P1 to P4, indicating higher conversion rates (see Figure S13 for detailed values). According to the acoustic field simulation results under different ultrasonic powers (Figures S14–S16), the increase in the polymerization conversion rate of the composite material is mainly attributed to the gradient‐increasing trend of acoustic intensity and acoustic pressure energy in the local region (the acoustic field distribution model and boundary conditions are described in the Supporting Information). As the ultrasonic power increases, the enhanced acoustic wave energy can effectively promote the polymerization reaction of the internal functional groups. The high intrinsic modulus of diamond particles [37] may also account for part of the overall elastic properties of the composite, whereas the acoustically regulated polymerization degree of the PDMS matrix offers an effective route for modulating the gradient properties.

Correspondingly, the low‐power region (P1), which has higher elasticity, undergoes greater deformation, whereas regions fabricated with higher powers (P2 → P4) exhibit increased stiffness and reduced compressibility. The curves in Figure 6d are consistent in trend with the simulated force–strain curves derived from the finite element simulations (Figure S12b,c). Quantitative analysis of the strain–force relationships reveals that under a load of 0.2 N (F4), the difference in strain between regions is significant (Δε_max_ = 9.17%, Figure 6e), confirming that precise gradient modulation is achievable through acoustic wave regulation.

### 2.4 Tactile Sensing Performance of Gradient Fingerprint Structures

The one‐step fabrication via graded acoustic wave energy enables a circumferentially graded structure that induces distinct mechanical responses across different regions, allowing the fingerprint structural layer to undergo larger contact area changes under the same applied load, thereby amplifying the force sensing response to the measured signal. To further exploit this amplification effect, flexible fingerprint structures with different gradient levels (G1, G2, G3, and G4) were used as functional layers to fabricate tactile sensors, while a pure PDMS layer (G4‐PDMS, fourth‐level gradient) was used as a control. As shown in Figure 7a, the assembled sensor was integrated into the fingertips of the robotic arm's fingers to improve its flexibility and practical adaptability.

**FIGURE 7:**
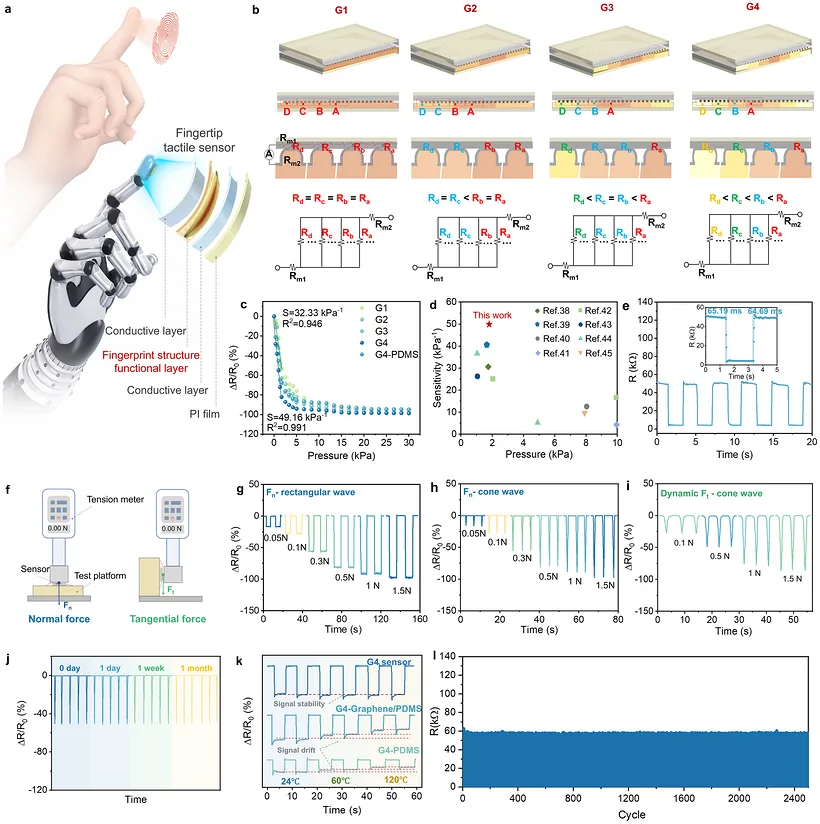
Sensing mechanism and performance of the fingertip sensor: (a) Assembly and integration of fingertip sensors; (b) Schematic illustration of the sensing mechanism; (c) Relative resistance variations of sensors with different gradient performances (G1–G4, G4‐PDMS); (d) Sensitivity comparison between the G4 sensor and a conventional PDMS‐based sensor; (e) Response and recovery times of the G4 sensor under a pressure of 2 kPa; (f) Illustration of tangential and normal loading methods; (g) Rectangular wave signal output; (h) Conical wave signals of the G4 sensor under pressures ranging from 0.05 to 1.5 N; (i) Dynamic tangential force response of the G4 sensor under pressures from 0.1 to 1.5 N; (j) Stability after storage for different durations; (k) Comparison of signal stability at 24°C, 60°C, and 120°C (G4, G4‐PDMS, and G4‐Graphene/PDMS); (l) Cyclic durability performance.

The sensing mechanism of the tactile sensor incorporating a circumferential gradient fingerprint‐structured functional layer is shown in Figure 7b. The total resistance (*R*) of the sensor is composed of three components: the resistance (*R*_*m*1_) of the upper conductive layer, the resistance (*R*_*m*2_) of the functional layer, and the total contact resistance (*R_t_*), which can be expressed as follows:

(1) $$R=R_{m1}+R_{m2}+R_{t}$$

*R_t_* can be calculated using Equation (2):

(2) $$\frac{1}{R_{t}}=\frac{1}{R_{a}}+\frac{1}{R_{b}}+\frac{1}{R_{c}}+\frac{1}{R_{d}}+\cdot \cdot \cdot$$

Each contact resistance is inversely related to the change in contact area. Thus, the individual contact resistances (*R_a_*,  *R_b_*,  *R_c_* and *R_d_*) can be expressed as follows:

(3) $$R_{i}=\frac{V/J}{A}\;$$

where *V* is the applied voltage, *J* is the current density, and *A* is the contact area.

Therefore, the change in contact resistance can be expressed as follows:

(4) $$\Delta R_{i}=V/J\;\times \left(\frac{1}{A_{1}}-\frac{1}{A_{0}}\right)$$

where *A*_0_ and *A*_1_ represent the contact areas before and after pressure application, respectively.

As illustrated in Figure 7b, for uniform‐performance sample G1, the contact area changes at points A, B, C, and D remain small under identical pressure, resulting in limited variation in contact resistance (*R_a_*,  *R_b_*,  *R_c_* and *R_d_*). In contrast, the four‐level gradient sample G4 exhibits progressively greater contact area changes from A to D because of elastic differences across the ridge structure, with the outer layers deforming more than the inner layers. This leads to significantly amplified variations in contact resistance. From a sensing perspective, a higher degree of gradient differentiation yields greater signal changes per unit pressure, improving responsiveness to subtle mechanical stimuli.

Figure 7c shows the resistance variation and sensitivity of the fabricated sensors (the full‐range sensitivity values are provided in Figure S17). Owing to the more substantial contact area changes in the G4 functional layer under the same load, the sensor demonstrates higher sensitivity and better linearity within the low‐pressure range (0–1.67 kPa). Specifically, G4 achieves a sensitivity (S) of 49.16 kPa^−1^, which is 52.06% greater than that of G1, with a linearity (R^2^) of up to 0.991. The presence of diamond particles further broadens the acoustic gradient modulation range, allowing G4 to outperform the pure PDMS gradient sensor (G4‐PDMS) by 20.24%. Compared with previously reported PDMS‐based sensors (typical sensitivity < 40 kPa^−1^) [38, 39, 40, 41, 42, 43, 44, 45], G4 exhibits superior performance within 0–2 kPa, confirming sufficient sensitivity for ultralow‐pressure detection (Figure 7d). This characteristic offers potential for initial contact detection and micro‐force feedback control in robotic fingertip sensing.

The dynamic response of the G4 sensor under transient pressure further demonstrates its high performance. As shown in Figure 7e, under an instantaneous load of 2 kPa, the response and recovery times are 65.19 and 64.69 ms, respectively, approximately half of those of G1 (Figure S18). Therefore, the G4 sensor was selected for subsequent performance evaluation and practical demonstrations.

As shown in Figure 7f–i, the G4 sensor exhibits excellent stability, reversibility, and repeatability under both tangential and normal loads. In terms of detection capability, its detectable force can be as low as 0.01 N (approximately 33.3 Pa), at which the sensor still maintains a stable signal response (Figure S19). Furthermore, the G4 sensor shows a hysteresis of 7.31%, indicating reversible signal behavior during the loading–unloading cycle (Figure S20). The long‐term durability of the sensor was also examined after storage at room temperature for 0, 1, 7, and 30 days. The relative resistance under a pressure of 1 kPa remained stable at about 49% (Figure 7j). Notably, the incorporation of diamond significantly enhances the thermal conductivity of the functional layer, allowing faster heat dissipation over the same operating duration (Figure S21). As a result, the G4 sensor displays more stable resistance signals without obvious drift at 24°C, 60°C, and 120°C, outperforming similar matrix‐based sensors (G4‐PDMS and G4‐graphene/PDMS) (Figure 7k). Moreover, cyclic loading tests further validated its excellent stability and durability, revealing no significant signal degradation (Figure 7l).

The unique circumferential gradient fingerprint design in tactile sensors enhances structural deformation under minimal loads, enabling more sensitive detection of multidirectional force distributions and facilitating fine texture recognition (Figure 8a). As shown in Figure 8b, the sensor responded rapidly and precisely to surfaces with different roughness levels on a standard texture plate (VDI 3400; Figure S22), generating distinct relative resistance outputs. Repeated cyclic tests confirm the excellent stability and consistency of the relative resistance signals (Figure 8c,d), with all measurement errors within 5%, meeting the standard tolerance range of commercial roughness testers (Table S1).

**FIGURE 8:**
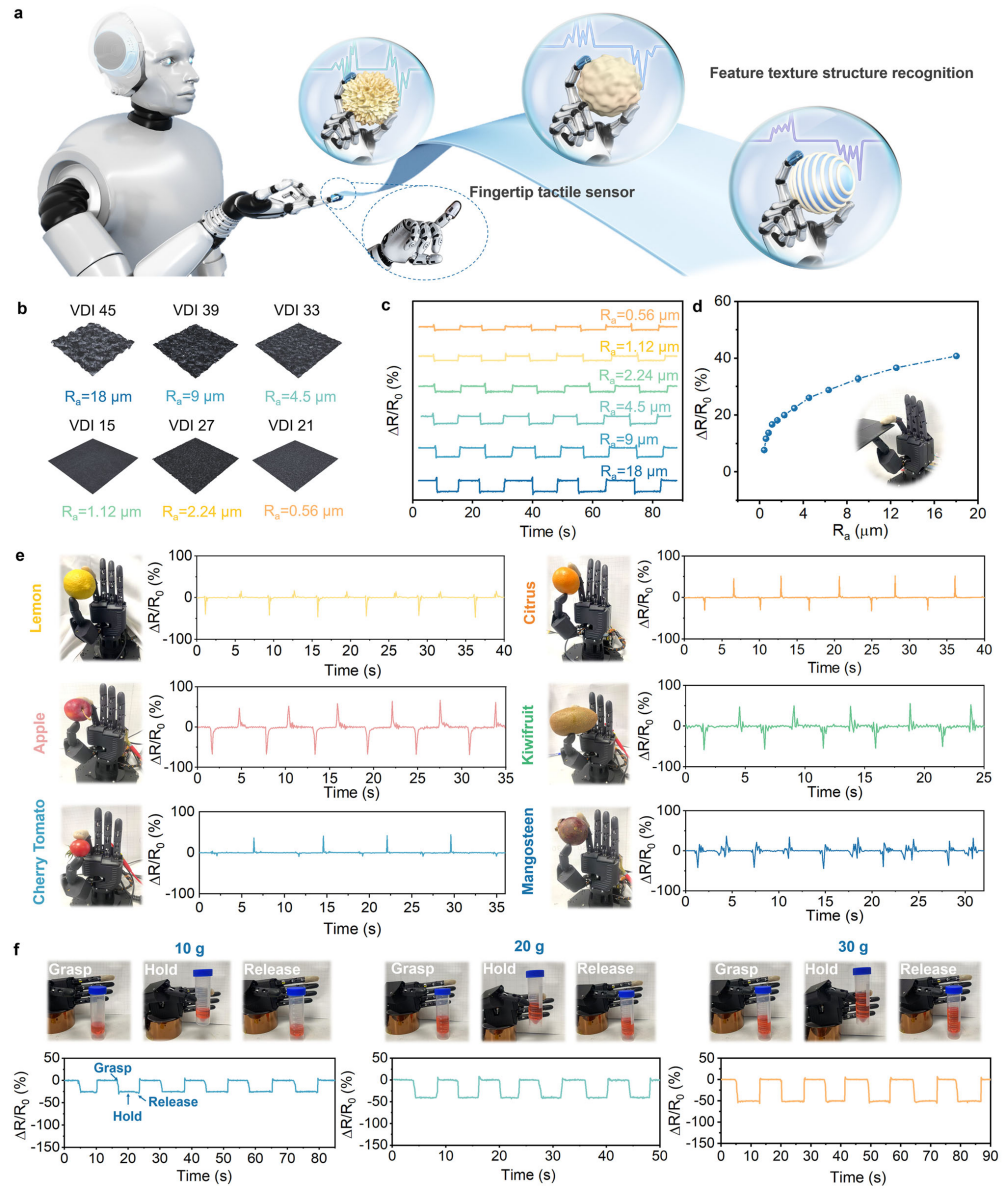
Fine tactile perception applications of the fingertip tactile sensor: (a) Schematic of the fingertip tactile sensor integrated on a robotic hand; (b) Surface morphology and roughness of a standard texture board (VDI 3400); (c) Response signals when contacting surfaces with varying roughness; (d) Variation in relative resistance signals with surface roughness; (e) Output signal curves when the robotic finger touches different fruits; (f) Response signals during continuous grasping, holding, and releasing of objects weighing 10, 20, and 30 g.

Given the unique surface texture patterns of different objects, the fingertip tactile sensor, when mounted on a robotic finger, enables precise texture recognition. Six fruits with representative surface features, such as lemon, citrus, apple, kiwi, cherry, and mangosteen, were selected for testing. As shown in Figure 8e, the robotic hand equipped with the fingertip sensor produces clearly distinguishable response waveforms while grasping each fruit, corresponding to their distinct surface textures. The sensor maintained highly consistent signal outputs over repeated grasping cycles, demonstrating excellent stability and reliability in texture identification.

To evaluate tangential force recognition under practical conditions, the sensor's ability to estimate object weight was tested using a robotic hand. The sensor was mounted on the inner surface of the robotic index finger. When the robotic hand grasped a liquid‐filled tube, the liquid weight generated a tangential force (F_t_) on the sensor. As shown in Figure 8f, when the total tube weight was adjusted to 10, 20, and 30 g, the fingertip tactile sensor consistently produced distinct resistance signals across multiple grasping and releasing cycles. As the liquid was gradually added in 1 g increments, the sensor accurately detected corresponding tangential force variations with a fast response and stable output, confirming its applicability for object weight estimation (Figure S23). Overall, as a fingertip tactile sensor designed for human–machine interactions, it demonstrates strong potential for automation tasks that traditionally rely on human tactile perception.

## 3 Conclusion

In this study, we reveal the mechanisms underlying acoustic‐induced curing and gradient property tuning in directed acoustic construction (DAC) technology. Acoustic‐induced curing arises primarily from synergistic physical, chemical, and mechanical effects, all of which can be reinforced by the incorporation of diamond particles. At the microscopic scale, the localized energy generated by the physical effects of acoustic cavitation induces acoustic curing, whereas the diamond particles provide multiple nucleation sites for cavitation bubbles. This intensifies the cavitation effect and enhances its distribution uniformity, promoting more frequent bubble nucleation, growth, and collapse cycles and thereby accelerating the localized curing rate. The essence of this acoustically induced curing is attributed to the chemical effects of free radicals. Intense ultrasonic shock waves at the diamond particle surface generate a higher concentration of reactive free radicals, and the reinforced acoustic chemical effect effectively enhances the polymerization and cross‐linking. Furthermore, the incorporation of diamond particles facilitates acoustic mechanical vibration‐induced molecular chain motion and collision, further increasing the degree of polymerization. The reinforcement of physical, chemical, and mechanical effects by diamond particles expands the tunable range of internal polymerization and cross‐linking via acoustic waves, thereby broadening the regulation window of gradient properties.

On this basis, the DAC‐fabricated fingerprint structures with circumferential gradient characteristics exhibit distinct local modulus variations, resulting in more pronounced gradient deformation under pressure. This structural response amplifies the signal variation per unit pressure, thereby improving the sensitivity to subtle mechanical stimuli. Within the low‐pressure range of 0–1.67 kPa, the sensor employing this fingerprint structure as the functional layer achieves a sensitivity (S) of up to 49.16 kPa^−1^ (exceeding the typical < 40 kPa^−1^ range for similar sensors), a linearity of 0.991, and a response time as short as 64.69 ms (about twice as fast as conventional designs). Furthermore, when integrated into a robotic finger, the sensor enables accurate detection of surface roughness, fine texture features, and object weight.

## 4 Experimental Section

### 4.1 DAC Gradient Fingerprint Structures

The composite material consisted of a polydimethylsiloxane matrix (PDMS; Dow Corning SYLGARD 184) reinforced with diamond particles (MBD‐4, 1–180 µm, 10 vol%, supplied by Henan Borui Co., Ltd., China). A micro/nano 3D printer (S230, Shenzhen Mofang New Material Technology Co., Ltd.) was used to fabricate a fingerprint‐like template (inner diameter: 200 µm; height: 250 µm), which was fixed to the inner wall of the outer tank. Diamond particles and PDMS were uniformly mixed, degassed under vacuum, and injected into the tank. An ultrasonic probe mounted on a six‐axis robotic arm followed a predefined elliptical trajectory to apply acoustic waves directly to the material. The region between the fingerprint‐patterned template and the inner wall of the outer tank was cured during sonication. After curing, the template was removed to obtain a flexible fingerprint microstructure layer. A low‐frequency ultrasonic transducer with an ultrasonic amplitude ranging from 0 to 11 µm was used, designated as A0–A5. A high‐frequency focused ultrasound transducer (frequency = 2 MHz; focal length ≈ 25 mm) was also employed, with an input power ranging from 5 to 17.5 W (designated P1–P4).

### 4.2 Assembly and Integration of the Tactile Sensor

A ridge‐shaped fingerprint‐structured diamond/PDMS composite material with circular elastic gradient properties was used as the functional layer of the sensor (G4, four‐level gradient; diamond particle size: 1 µm; diamond content: 10 vol%), while a pure PDMS layer with the same gradient configuration (G4‐PDMS, four‐level gradient) served as the control. A conductive film composed of a graphene/PDMS composite was placed atop the functional layer and coated with a graphene‐based conductive layer. Electrodes made of conductive fabric were affixed to both the upper and lower surfaces of the conductive and functional layers using silver paste to ensure proper electrical contact. The entire sensor was then encapsulated with a PI film to complete the assembly.

To enhance adaptability and flexibility, the sensor was embedded in a customized finger sleeve fabricated by inverse molding, ensuring a snug fit on the user's finger. Additionally, the sensor can be integrated onto a robotic finger. Using control software, the robotic hand can be programmed to grasp objects or scan surfaces with different roughness levels, enabling the assessment of fine tactile perception capabilities under practical conditions.

### 4.3 Characterization and Measurements

The temporal evolution of ultrasonic cavitation bubbles was captured using a Photron Fastcam SA3 high‐speed camera at 150 000 frames per second, with an image resolution of 672 × 448 pixels. To detect hydroxyl radical signals, 5,5‐dimethyl‐1‐pyrroline N‐oxide (DMPO) was used as a radical trapping agent. The concentration and spatial distribution of free radicals in the system were analyzed by electron spin resonance (EPR) spectroscopy. The relative polymerization conversion rate (*C_t_*) was determined using FTIR spectroscopy, with a sample size of 10 mm × 10 mm × 2 mm (length × width × thickness). For each test, five samples were used, and the mean value was taken as the measured value after excluding the maximum and minimum values. The main reactive functional groups in the PDMS matrix are C═C and Si─H bonds, while the peak area of the methyl functional group at 1260 cm^−1^ remains constant and serves as an internal reference. The relative polymerization conversion rate was determined from the change in the ratio of the peak areas of the reactive functional groups to that of the methyl group before and after the reaction, as defined by Equation (5):

(5) $$C_{t}=\frac{{\left(A_{x}-A_{1260}\right)}_{t}-{\left(A_{x}-A_{1260}\right)}_{0}}{{\left(A_{x}-A_{1260}\right)}_{0}}\;\times 100\%$$

where *A*_1260_ is the peak area of the methyl group, *A_x_* represents the peak area of the reactive functional group, (*A_x_* − *A*_1260_)_*t*_ denotes the difference after ultrasonic treatment, and (*A_x_* − *A*_1260_)_0_ is the initial difference. The degree of cross‐linking (*C_L_*) is defined as the percentage of cross‐linked molecules relative to the total molecular content of the matrix. Since cross‐linked products are insoluble in n‐hexane, n‐hexane was used as the extraction solvent for determining the extent of cross‐linking.

The mass ratio of cross‐linked products before and after extraction represents the degree of cross‐linking:

(6) $$C_{L}=\frac{M_{t}}{M_{0}}\;\times 100\%$$

where *M*_0_ and *M_t_* are the masses of the diamond/PDMS composite before and after extraction, respectively. The cross‐linking density represents the number of cross‐linking points per unit volume, i.e., the quantity of cross‐linked bonds within a defined volume. The cross‐linking density (D) of the composites was determined using the equilibrium swelling method. The samples were first immersed in acetone to remove any uncured material and then transferred into toluene. The solvent was replaced every 24 h, and the samples were weighed repeatedly until their mass stabilized, indicating equilibrium swelling. The cross‐linking density was calculated using Equation (7) [46, 47]:

(7) $$\ln\left(1-\varphi \right)+\varphi +\chi \varphi =-D\times V_{1}\times \varphi^{\frac{1}{3}}$$

Where *D*, *V*_1_, *χ*, and *φ* represent the cross‐linking density, molar volume of toluene (106.113 mL·mol^−1^), Flory–Huggins interaction parameter, and polymer volume fraction in the swollen gel, respectively. The volume fraction φ was calculated using Equation (8):

(8) $$\varphi =\frac{m_{0}/\rho_{2}}{m/\rho_{1}-m_{0}\left(1/\rho_{2}-1/\rho_{1}\right)}\;$$

Here, ρ_1_ is the solvent density (0.867 g/mL), ρ_2_ is the density of the PDMS, and *m*_0_ and *m* denote the sample masses before and after swelling, respectively.

The deformation behavior of elastic microstructures under simultaneous loading was recorded using a Photron Fastcam SA3 high‐speed camera operating at 10 000 fps. The contact width at each deformation stage was measured with ImageJ software. In situ mechanical tests were conducted to assess the response of the microstructures, with the indenter moving at a constant loading rate of 2 µm·s^−1^.

The fabricated sensor was mounted on a pressure testing platform, and its electrodes were connected to a Keithley 2450 digital source meter. A normal load (0–20 kPa; each 0.1 N increment corresponding to 0.33 kPa) was applied using an Aidebao push–pull tester to evaluate the normal force response. A tangential load was applied through the sidewall of the force probe to assess the shear response. The sensitivity (S) was defined as the slope of the curve of the relative resistance change versus the applied pressure.

Temperature dissipation under different conditions was monitored using an infrared thermal imager (A615, FLIR). The thermal diffusivity (α) of different functional layer materials was measured using a thermal conductivity analyzer (LFA467, Netzsch, Germany). The specific heat capacity (*c_p_*) was determined via differential scanning calorimetry (DSC3, METTLER TOLEDO). The thermal conductivity (*k*) was then calculated according to Equation (9):

(9) $$k=\alpha \times c_{p}\times \rho$$

where α, *c_p_*, and ρ represent the thermal diffusivity, specific heat capacity, and material density, respectively.

## Author Contributions

**Dandan Wu**: investigation, data curation, visualization, writing – original draft, Writing – review and editing, methodology. **Lijuan Zheng**: methodology. **Guang Yao**: methodology. **Zijun Liu**: visualization, investigation. **Liyan Luo**: visualization. **Cile Liang**: investigation. **Chengyong Wang**: conceptualization, methodology, supervision, writing – review and editing, funding acquisition, project administration.

## Conflicts of Interest

The authors declare no conflicts of interest.

## Supporting information




**Supporting File 1**: advs78376‐sup‐0001‐SuppMat.docx.

**Supporting File 2**: advs78376‐sup‐0002‐MovieS1.mp4.

**Supporting File 3**: advs78376‐sup‐0003‐MovieS2.mp4.

**Supporting File 4**: advs78376‐sup‐0004‐MovieS3.mp4.

## Data Availability

Research data are not shared.
