# Supplementary material for: Mechanism of Direct Acoustic Construction and Gradient Properties Tuning
Source: Adv Sci (Weinh). 2026 Oct 20:e78376. Online ahead of print. doi: 10.1002/advs.78376 (PMC13683983; doi:10.1002/advs.78376)
Supplement: Supplementary file 1 [file ADVS-9999-e78376-s003.docx]

**Supplementary Information**

**Mechanism of Direct Acoustic Construction and Gradient Properties Tuning**

Dandan Wu ^a,b,c,d^, Lijuan Zheng ^a,b,c,d^, Guang Yao ^a,b,c,d^, Zijun Liu ^a,b,c,d^, Liyan Luo ^a,b,c,d^, Cile Liang ^a,b,c,d^, Chengyong Wang ^a,b,c,d, *^

^a^School of Electromechanical Engineering, Guangdong University of Technology, Guangzhou 510006, China.

^b^State Key Laboratory for High Performance Tools, Guangdong University of Technology, Guangzhou 510006, Guangdong, China.

^c^Guangdong Provincial Key Laboratory of Minimally Invasive Surgical Instruments and Manufacturing Technology, Guangdong University of Technology, Guangzhou 510006, Guangdong, China.

^d^Smart Medical Innovation Technology Center, Guangdong University of Technology, Guangzhou 510006, Guangdong, China.

*Corresponding author: cywang@gdut.edu.cn (Chengyong Wang)

**Table of Contents**

[Supplementary Notes	4](#_Toc241075185)

[Supplementary Note 1: Mechanisms of Acoustic Curing and Reinforcement in DAC	4](#_Toc241075186)

[Supplementary Figure 1 | Evolution of bubble clouds under different ultrasound amplitudes (A1–A5) captured via high-speed photography.	4](#_Toc241075187)

[Supplementary Figure 2 | Bubble cloud evolution in diamond/PDMS composites containing different particle sizes.	5](#_Toc241075188)

[Supplementary Figure 3 | Cavitation bubble formation on the surface of diamond particles captured by high-speed photography.	6](#_Toc241075189)

[Supplementary Figure 4 | Size variation of cavitation bubbles over multiple cycles.	7](#_Toc241075190)

[Supplementary Figure 5 | Evolution of cavitation bubbles at different nucleation sites.	8](#_Toc241075191)

[Supplementary Figure 6 | Acoustic chemical effects of composites reinforced with different particles.	9](#_Toc241075192)

[Supplementary Figure 7 | Effect of the acoustic mechanical force application time on the cross-linked networks in the composites.	10](#_Toc241075193)

[Supplementary Note 2: Acoustic Tuning of Gradient Properties in Fingerprint Structures	12](#_Toc241075194)

[Supplementary Figure 8 | Design and DAC of circumferential-gradient fingerprint structures.	12](#_Toc241075195)

[Supplementary Figure 9 | Compressive properties of micropillars fabricated at different ultrasonic powers.	13](#_Toc241075196)

[Supplementary Figure 10 | Deformation evolution of surface microstructure captured by high-speed photography (samples G2 and G3)	14](#_Toc241075197)

[Supplementary Figure 11 | Boundary conditions of the finite element model	14](#_Toc241075198)

[Supplementary Figure 12 | Finite element simulation of the deformation characteristics of the microstructures.	16](#_Toc241075199)

[Supplementary Figure 13 | Effects of ultrasonic power on polymerization.	17](#_Toc241075200)

[Supplementary Figure 14 | Boundary conditions of the finite element model	17](#_Toc241075201)

[Supplementary Figure 15 | Acoustic intensity distributions under different ultrasonic powers.	19](#_Toc241075202)

[Supplementary Figure 16 | Acoustic pressure distributions under different ultrasonic powers.	19](#_Toc241075203)

[Supplementary Note 3: Tactile Sensing Performance of Gradient Fingerprint Structures	20](#_Toc241075204)

[Supplementary Figure 17 | Sensitivity of the G4 sensor over other pressure ranges.	20](#_Toc241075205)

[Supplementary Figure 18 | Response speed of tactile sensors.	20](#_Toc241075206)

[Supplementary Figure 19 | Response curve of the G4 sensor under a low pressure of 0.01 N.	21](#_Toc241075207)

[Supplementary Figure 20 | Hysteresis curve of the G4 sensor.	21](#_Toc241075208)

[Supplementary Figure 21 | Characterization of high-temperature stability and fatigue resistance.	22](#_Toc241075209)

[Supplementary Figure 22 | Photograph of the standard texture plate and corresponding roughness values used for roughness recognition with the fingertip tactile sensor.	23](#_Toc241075210)

[Supplementary Table 1 Sensor recognition values and actual values of roughness	23](#_Toc241075211)

[Supplementary Figure 23 | Signal curves corresponding to liquid weight changes in a tube grasped by the robotic hand	24](#_Toc241075212)

[Supplementary References	25](#_Toc241075213)

# Supplementary Notes

# Supplementary Note 1: Mechanisms of Acoustic Curing and Reinforcement in DAC

## **Supplementary Figure 1 |** Evolution of bubble clouds under different ultrasound amplitudes **(A1–A5)** captured via high-speed photography.

To investigate the relationship between acoustic cavitation and cured material formation, different ultrasonic amplitudes (45 kHz) were applied to the PDMS matrix, and bubble cloud evolution was recorded using high-speed photography. The applied ultrasound amplitudes were 7.7 μm, 8.2 μm, 8.8 μm, 9.4 μm, 10.2 μm, and 11 μm, designated A1, A2, A3, A4, and A5, respectively. As ultrasonic waves propagate through the liquid, alternating compression and rarefaction cycles generate positive and negative pressure regions. High negative pressure facilitates bubble nucleation. As shown in Supplementary Figure 1, small cavitation bubbles emerge under ultrasound and expand rapidly with prolonged exposure. With increasing ultrasound amplitude (A1 to A5), the cavitation bubble cloud size progressively increases over time.

## **Supplementary Figure 2 |** Bubble cloud evolution **in diamond/PDMS composites** containing **different particle sizes.**

As shown in Supplementary Figure 2, under identical ultrasonic durations, the bubble cloud size increased markedly in D60 and D120, whereas that in D180 remained comparable to that in D0. Moreover, smaller particles promoted a wider spatial distribution of cavitation bubbles. After 42 ms of ultrasound exposure, the cross-sectional area of the bubble cloud in the D60 sample reached 8.28 mm^2^, whereas it was only 2.21 mm^2^ in pure PDMS (Supplementary Figure 1).

## **Supplementary Figure 3 | Cavitation bubble formation on the surface of diamond particles captured by high-speed photography**.

To further clarify the role of the particles in the cavitation behavior, 60 μm diamond particles were fixed at the bottom of the PDMS tank to facilitate clear observation of bubble nucleation on their surfaces. As shown in Supplementary Figure 3, minute bubbles began to appear on the diamond surfaces after 3.5 ms of ultrasound exposure. By 7 ms, cavitation bubbles about 10 μm in diameter were uniformly distributed on the particle surfaces. These results confirm that diamond particles create favorable conditions for cavitation nucleation. Surface roughness, microdefects, or specific chemical surface characteristics may provide additional nucleation sites ^[1-3]^. Furthermore, at a given volume fraction, smaller particles supply a greater number of evenly distributed nucleation sites, increasing the bubble population and density ^[4]^. The enhanced cavitation effect generates stronger shock waves and higher localized energy, which accelerates and improves the curing efficiency.

## Supp**lementary Figure 4 | Size variation of cavitation bubbles over multipl**e cycles.

Furthermore, Figure 3 presents the evolution of the cavitation behavior on the surface of a single particle. Taking the particle marked by the red circle as an example, three complete nucleation–growth–collapse cycles were recorded. The variation in the bubble size across multiple cycles is shown in Supplementary Figure 4, with blue and red markers indicating the maximum and minimum diameters, respectively, measured in repeated experiments. During the first cycle, alternating positive and negative acoustic pressures in the liquid induced vibration and gradual growth of bubble nuclei on the diamond surface. A stable bubble of 45.5 μm formed at 3.6 ms, followed by collapse at 3.8 ms because of ultrasonic compression and increased internal pressure, completing the cycle within 4 ms.

During the second cycle, nucleation occurred more rapidly, with a stable bubble forming within 0.8 ms at an average size of 23.5 μm and collapsing after 0.4 ms. At 5.6 ms, a third cycle began, and a new cavitation bubble reappeared at the same nucleation site, with its average size further reduced to 11.5 μm.

## **Supplementary Figure 5 | Evolution of cavitation bubbles at different nucleation sites.**

**(a)** Evolution of cavitation bubbles in the PDMS liquid; **(b)** Evolution of cavitation bubbles on the surface of SiO₂ particles; **(c)** Nucleation time; **(d)** Single cycle duration.

To further validate the enhancing effect of diamond particles on the acoustic cavitation behavior, SiO₂ particles with comparably high acoustic impedance were selected for comparison. High-speed photography images of homogeneous nucleation in the PDMS liquid are shown in Supplementary Figure 5a, and the evolution of heterogeneous nucleation on the SiO₂ surface is shown in Supplementary Figure 5b. In both cases, only a single bubble oscillation cycle was observed. The formation times of bubbles with a size of approximately 45 μm at different nucleation sites (diamond particles, SiO₂ particles, and liquid medium with the same particle size of 60 μm) and the duration of a single cycle are shown in Supplementary Figure 5c and d, respectively. Statistical analysis, averaged over 20 bubbles of similar size, revealed that heterogeneous nucleation on particle surfaces required less time than homogeneous nucleation in the liquid (Supplementary Figure 5d). Furthermore, compared with the introduction of SiO₂ particles, the introduction of diamond particles shortened the nucleation time by an additional 4.44 ms. Moreover, compared with those of the PDMS liquid and SiO₂ particles, the complete cycle times of bubble nucleation, growth, and collapse on the diamond particles were shorter, by 71.96% and 51.73%, respectively. This improvement is attributed to the more complex geometry and larger surface area of the diamond particles. Additionally, the higher acoustic impedance of diamond than that of SiO₂ may induce stronger ultrasonic reflections (diamond: ~60.8 × 10⁶ kg/m²·s^[5]^; SiO₂: ~13 × 10⁶ kg/m²·s^[6]^), concentrating energy at the particle surface and thereby promoting bubble nucleation and growth.

## Supplementary Figure 6 | Acoustic chemical effects of composites reinforced with different particles.

**(a)** FTIR spectra; **(b)** Radical signals (amplitude: A1).

The curing behavior driven by physical acoustic effects originates from chemical acoustic effects at the microscopic level. The rapid oscillation of ultrasonic waves and the resulting cavitation generate extreme local thermodynamic conditions, causing molecular dissociation into highly active radicals such as hydroxyl (·OH), hydrogen (H·), and hydrogen peroxide (H_2_O_2_) ^[7, 8]^. These radicals, with unpaired electrons and high reactivity, initiate cross-linking and polymerization reactions in resin systems such as PDMS.

The enhancement effect of diamond particles on reactive free radicals and the resulting polymerization are shown in Figue 4. Furthermore, we compared the acoustic chemical effects of composite materials reinforced with different particles. As shown in Supplementary Figure 6a, both the SiO₂ particles and the diamond particles exhibit absorption bands at 1641 cm^-1^ and 3400 cm^-1^, respectively. The prominent absorption peak at 3400 cm^-1^ corresponds to the characteristic absorption band of hydroxyl groups, indicating the presence of hydrophilic hydroxyl functional groups on the surfaces of both particles. However, the diamond particles clearly possess a higher density of hydroxyl groups on their surface. Therefore, under ultrasonic excitation, they exhibit stronger free radical signals, confirming that diamond particles can more effectively enhance the effects of acoustic chemicals (Supplementary Figure 6b).

## **Supplementary Figure 7 | Effect of the acoustic mechanical force application time on the cross-linked networks in the composites**.

**(a)** Cross-linking density; **(b)** Cross-linking degree;**(c)** Illustration of cross-linking of diamond /PDMS composites under acoustic mechanical forces.

The application of an acoustic mechanical force also strengthens molecular chain cross-linking within the composite system. As shown in Supplementary Figures. 7a–b, in the composite reinforced with 1 μm diamond particles, extending the acoustic mechanical action from 60 s to 240 s increased the cross-linking density by 30.01%, corresponding to a strengthening effect 119.53% greater than that in pure PDMS. This remarkable increase is attributed to the ability of the diamond particles to disperse and amplify mechanical forces. In liquid media, the mismatch in mechanical properties at the particle–matrix interface induces wave scattering, leading to acoustic momentum redistribution and the generation of localized mechanical forces that drive particle migration ^[9-11]^. In contrast, in solid media, particle migration is restricted, which can lead to wave reflection or resonance. These mechanisms may cause localized force reversal or substantial amplification ^[12]^. The improved spatial dispersion of the acoustic mechanical forces facilitated by the diamond particles promotes molecular chain movement and collisions, thereby accelerating polymerization and enhancing cross-linked network formation (Supplementary Figure 7c).

# Supplementary Note 2: Acoustic Tuning of Gradient Properties in Fingerprint Structures

## **Supplementary Figure 8 | Design and DAC of circumferential-gradient fingerprint structures**.

Supplementary Figure 8 displays ridge-shaped fingerprint structures with varying gradient characteristics. Sample G1, which was fabricated under uniform acoustic energy (P4), exhibits homogeneous properties. Applying different acoustic energies to the inner (P3) and outer (P4) regions yields a dual-gradient sample (G2). Sequentially applying three graded energies (P2, P3, and P4) from the center outward produces a triple-gradient elastic sample (G3). Finally, four concentric curing layers with progressively increasing acoustic energies from P1 to P4 generate a four-level gradient sample (G4).

## **Supplementary Figure 9 | Compressive properties of micropillars fabricated at different ultrasonic powers**.

**(a)** Stress‒strain curves; **(b)** Elastic modulus.

To obtain a surface microstructure array with a distinct elasticity gradient, four ultrasonic power levels of appropriate intensity were selected. Micropillar arrays with identical dimensions (diameter: 200 μm; height: 250 μm) were fabricated under varying ultrasonic power levels—P5, P7.5, P10, P12.5, P15, and P17.5. The mechanical properties were evaluated using in situ compression tests, with the loading process recorded at 1 mm·min^–1^. The stress‒strain curves of the micropillars are shown in Supplementary Figure 9a, where stress is defined as the applied load per unit area and strain is defined as the relative displacement. Increased ultrasonic power enhances micropillar stiffness. At 80% strain, the P17.5 sample has the highest strength (6.77 MPa), whereas the P5 sample reaches only 0.91 MPa, indicating that a higher power requires a greater compressive force for equivalent deformation. The stress‒strain curves of P7.5 and P10, as well as P12.5 and P15, show similar profiles with minor differences in elastic modulus (Supplementary Figure 9b). Based on these findings, P5, P10, P12.5, and P17.5 were selected as the four designated power levels (denoted as P1–P4) for subsequent DAC fabrication to achieve clear elastic differentiation on a single surface.

## Supplementary Figure 10 | Deformation evolution of surface microstructure captured by high-speed photography (samples G2 and G3)

For the uniform-performance functional layer G1, as the pressure increases from F1 (0.15 N) to F2 (0.3 N), the deformation at points A, B, C, and D increases uniformly, and the cross-sectional contact lengths L_a_, L_b_, L_c_, and L_d_ exhibit consistent changes. When the ultrasound power is increased to two higher levels (P3 and P4), the corresponding two-level elastic gradient functional layer G2 begins to exhibit gradient changes in contact area. Under a pressure of F2, L_a_ and L_b_ are approximately 149 μm, while L_c_ and L_d_ increase to 180 μm. Furthermore, as the degree of elastic gradient increases, the magnitude of contact area variation becomes more pronounced. For the three-level elastic gradient sample G3, the contact point cross-sectional lengths follow the order L_a_ < L_b_ = L_c_ < L_d_ (Supplementary Figure 10).

Finite Element Simulation of Microstructure Deformation Behavior:

## Supplementary Figure 11 | Boundary conditions of the finite element model

To investigate the deformation characteristics of microstructures with uniform and gradient properties, we further established a model of the microstructure deformation process using the COMSOL finite element simulation software. The dimensions of the constructed physical model are consistent with those of the experimentally prepared samples, both being 200 × 250 μm (width × height). Under quasi-static compression conditions, the mechanical response of the microstructure is described based on the theory of finite deformation solid mechanics ^[13, 14]^. In this case, the microstructure satisfies the following nonlinear static equilibrium equation:

$\nabla\cdot\sigma=0$	(1)

where $\sigma$ is the Cauchy stress tensor. To describe the deformation during compression, the deformation gradient tensor is defined using the finite strain kinematic relationship:

$F=I+\nabla u$	(2)

where $u$ is the displacement vector and $I$ is the identity tensor. The Cauchy deformation tensor is further defined as $C=F^{T}F$, and the volume change is characterized by $J=detF$. Due to the pronounced nonlinear elasticity and strain hardening behavior of the material under large compressive deformation, a third-order Yeoh model is adopted to describe the constitutive response of the microstructure. Its total strain energy density function is expressed as:

${W=W}_{iso}+W_{vol}$	(3)

The isochoric strain energy density function is expressed as:

$W_{iso}=C_{1}(\overline{I_{1}}-3)+C_{2}{(\overline{I_{1}}-3)}^{2}+C_{3}{(\overline{I_{1}}-3)}^{3}$	(4)

Where $\overline{I_{1}}$ is the first invariant corresponding to isochoric deformation, and $C_{1}、C_{2}$ and $C_{3}$ are Yeoh model parameters obtained by fitting the experimental compression curves. Meanwhile, considering the nearly incompressible nature of the material, the volumetric strain energy can be expressed as:

$W_{vol}=\frac{1}{2}K{(J-1)}^{2}$	(5)

where $K$ is the bulk modulus. The material stress is obtained by differentiating the strain energy function with respect to the deformation tensor:

$S=2\frac{\partial W}{\partial C}$	(6)

which is further converted to the Cauchy stress: $\sigma=\frac{1}{J}\mathrm{FS}F^{T}.$

The above process is solved through finite element simulation to obtain the displacement field $u\left( x , y \right)$, thereby yielding the deformation magnitude of the microstructure under applied loads.

## Supplementary Figure 12 | Finite element simulation of the deformation behavior of the microstructures.

**(a)** Deformation of microstructures with different gradient properties (G1 and G4); **(b)** Simulated force–strain curves of the local regions (Sim. P1–P4) in the G4 microstructure; **(c)** Comparison between the experimental and simulated strain values of the local regions in the G4 microstructure under F2 loading.

The Supplementary Figure 12a shows the simulation results of the deformation process for the homogeneous microstructure G1 and the four-level gradient microstructure G4 (fabricated at powers P1–P4, respectively). The force–strain curves derived from the simulation results (Supplementary Figure 12b) indicate that the microstructures fabricated at different powers exhibit distinct gradient deformation characteristics, with their strain distributions showing a gradient trend. The force–strain curves obtained from the simulations show a trend consistent with the experimental results in Figure 6d. Notably, however, under the same applied load, the simulated strain values are slightly lower than the experimental values. For instance, under the same pressure F2, the simulated strain values of the microstructures fabricated at powers P1–P4 are approximately 4% lower than the experimental values (Supplementary Figure 12c). This may be attributed to the fact that simulation models are based on idealized boundary conditions and material properties, whereas the elastomeric material in actual experiments often exhibits strain softening or viscoelasticity, resulting in slightly larger actual deformation than predicted by simulation.

## **Supplementary Figure 13 | Effects of ultrasonic power on polymerization**.

The conversion rates of the reactive functional groups under the selected powers P1–P4 are shown in Supplementary Figure 13. As the power increased from P1 to P4, the conversion rates of vinyl and Si-H increased by 82.5% and 50.07%, respectively.

Finite Element Simulation of Acoustic Field Distribution Characteristics:

## **Supplementary Figure 14 |** Boundary conditions of the finite element model

To further analyze the acoustic field distribution characteristics under different ultrasonic powers (P1-P4), a physical model was established using the COMSOL Multiphysics finite element software. The acoustic field simulations were carried out at room temperature (25 °C) and standard atmospheric pressure, with the solution tank dimensions set to be consistent with those used in the experiments, i.e., 85 × 110 mm (height × width). By coupling the pressure acoustics, frequency domain, and acoustic-structure boundary modules, the acoustic intensity and acoustic pressure distributions under different ultrasonic powers were predicted. The medium layer in the solution tank was a diamond-reinforced composite material (particle size: 1 μm, content: 10 vol%).

In the established physical model, the acoustic field in the PDMS liquid medium is described by the Helmholtz equation ^[15]^：

$\nabla\cdot\left（ -\frac{1}{\rho_{c}}\nabla p \right）-\frac{\omega^{2}p}{\rho_{c}c_{c}^{2}}=0$	(7)

where $p$, $\rho_{c}$, ω, and $c_{c}$ are the acoustic pressure, density, angular frequency, and speed of sound, respectively. Diamond particles are introduced into the matrix as a reinforcing phase and are treated as linear elastic solids. Bidirectional coupling between the acoustic field and the solid domain is achieved through the acoustic–structure interface. The acoustic pressure generated in the liquid acts on the particle surfaces and exerts an inward normal load, which can be calculated by Eq. 2. In addition, the vibration of the particle surfaces reacts on the acoustic field, satisfying the normal acceleration continuity condition (Eq. 3).

$F_{A}=-n_{s}p$	(8)

$a_{fluid,n}=a_{solid,n}$	(9)

where $F_{A}$ is the acoustic load per unit area, $n_{s}$ is the outward normal vector of the solid, and $a_{fluid,n}$ and $a_{solid,n}$ are the normal accelerations of the fluid and the solid, respectively. This coupling is implemented through the acoustic–structure interface boundary condition：

$-n\left( -\frac{1}{\rho_{c}}\left( \nabla p-q_{d} \right) \right)=-n\cdot u_{tt}$	(10)

where $q_{d}$ is the dipole source term, $n$ is the outward normal vector of the acoustic domain, and $u_{tt}$ is the vibrational acceleration of the particle surface.

## **Supplementary Figure 15 |** Acoustic intensity distributions under different ultrasonic powers.

**(a)** P1; **(b)** P2; **(c)** P3; **(d)** P4

## **Supplementary Figure 16 |** Acoustic pressure distributions under different ultrasonic powers.

**(a)** P1; **(b)** P2; **(c)** P3; **(d)** P4

Supplementary Figures 15 and 16 present the acoustic intensity and acoustic pressure distribution characteristics under ultrasonic powers P1–P4, respectively. It can be observed that the acoustic intensity at the center of the focused ultrasound focal point exhibits a gradient increase as the ultrasonic power increases. When the power increases from P1 to P4, the acoustic intensity at the center point rises from 1.59×10⁶ W/m³ to 3.7×10⁶ W/m³. In addition, when ultrasound propagates in a liquid medium, it forms an alternating positive–negative pressure cycle, generating compressional and rarefactional waveforms. As the input power increases, the amplitude of the periodic positive–negative pressure induced by the acoustic wave also increases, with the maximum acoustic pressure rising from 1.2 MPa to 1.83 MPa. As this gradient acoustic energy continuously moves along the radial direction, it induces the formation of a gradient polymerization structure and thus fingerprint structures with varying elastic moduli.

# Supplementary Note 3: Tactile Sensing Performance of Gradient Fingerprint Structures

## **Supplementary Figure 1**7 **|** Sensitivity of the G4 sensor over other pressure ranges.

## **Supplementary Figure 18 | Response speed of tactile sensors**.

**(a)** G1; **(b)** G2; **(c)** G3; **(d)** G4-PDMS.

The dynamic response and recovery curves of the sensors with different gradient characteristics under an instantaneous pressure of 2 kPa are shown in Supplementary Figure 18. The sensor with a uniform functional layer (G1) exhibits a relatively slow response to loading, with recorded response and recovery times of 141.39 ms and 127.25 ms, respectively. As the gradient degree of the functional layer increases, the sensor response speed improves progressively. For example, the G4 sensor, possessing the highest gradient level, achieves response and recovery times of 65.19 ms and 64.69 ms, respectively—about twice as fast as those of the G1 sensor. Moreover, owing to the expanded tunable range of gradient properties introduced by diamond particles, compared with the G4-PDMS sensor, the G4 sensor exhibits a more distinct gradient variation from the interior to the exterior of its functional layer, thereby further increasing the response speed.

## Supplementary Figure 19 | Response curve of the G4 sensor under a low pressure of 0.01 N.

## Supplementary Figure 20 | Hysteresis curve of the G4 sensor.

Supplementary Figure 19 presents the response curve of the G4 sensor under a pressure of 0.01 N (approximately 33.3 Pa), demonstrating its stable response at low pressure. In addition, Supplementary Figure 20 shows the relationship between the applied pressure and the relative change in resistance, from which the hysteresis can be calculated as:

$\gamma=\frac{{\Delta H}_{max}}{Y_{Fs}}\times100\%$	(11)

where ${\Delta H}_{max}$represents the maximum vertical difference between the loading and unloading curves, and $Y_{Fs}$ is the full-scale output of the sensor, i.e., the relative change in resistance when the applied pressure reaches its maximum value. The hysteresis of the G4 sensor is calculated to be 7.31%.

## **Supplementary Figure 21 | Characterization of high-temperature stability and fatigue resistance.**

(a) Infrared thermal images of sensors with PDMS, graphene/PDMS, and diamond/PDMS functional layers at different operating temperatures; (b) Cooling temperature curves after heating to 120 °C; (c) Thermal conductivity of three functional layers.

To assess thermal response behavior, sensing tests were conducted by heating the sensors on a temperature-controlled plate, followed by comparison with sensors utilizing conventional functional layers (pure PDMS and graphene/PDMS composites). All reference sensors were fabricated with identical dimensions and structural parameters as the G4 sensor. The infrared thermal images captured at 0, 15, 45, and 75 s are shown in Supplementary Figure 21a. The diamond/PDMS composite demonstrates a significantly faster cooling rate (Supplementary Figure 21b), which is consistent with its high thermal conductivity, which is nearly 17 times greater than that of pure PDMS (Supplementary Figure 21c).

## Supplementary Figure 22 | Photograph of the standard texture plate and corresponding roughness values used for roughness recognition with the fingertip tactile sensor.

## Supplementary Table 1 Sensor recognition values and actual values of roughness

| No. of specimen | 1 | 2 | 3 | 4 | 5 | 6 |
| --- | --- | --- | --- | --- | --- | --- |
| Standard value R_a_ (μm) | 0.45 | 1 | 2 | 3.2 | 7 | 14 |
| ΔR/R_0_ | 0.0952 | 0.1213 | 0.1954 | 0.2283 | 0.3050 | 0.3767 |
| Measured value Ra (μm) | 0.4683 | 1.0325 | 2.0721 | 3.2911 | 7.1821 | 14.3109 |
| Absolute error | 0.0183 | 0.3245 | 0.0721 | 0.0911 | 0.1821 | 0.3109 |
| Relative error | 4.07% | 3.25% | 3.61% | 2.85% | 2.60% | 2.22% |

To evaluate its tactile discrimination performance, the fingertip sensor was used to identify the surface roughness of a standard texture plate (VDI 3400) with varying R_a_ values, as shown in Supplementary Figure 22. Mounted on a robotic hand, the sensor tested multiple standard surfaces and responded accurately and rapidly upon contact, generating distinct relative resistance signals corresponding to different roughness levels. The calculated measurement errors remained within 5%, which is consistent with the accuracy range of conventional roughness testers (Supplementary Table 1).

## Supplementary Figure 23 | Signal curves corresponding to liquid weight changes in a tube grasped by the robotic hand

With the gradual addition of liquid in 1 g increments, the sensor accurately tracked the resulting tangential force variations, resulting in a fast response and stable output. The recorded waveforms exhibited near symmetry during both the filling and draining stages (Supplementary Figure 23).

# Supplementary References

[1]	Q. Zhang,H. Xue,H. Zhang, et al., "Enhanced thrombolytic effect induced by acoustic cavitation generated from nitrogen-doped annealed nanodiamond particles," *Ultrason. Sonochem.* 99, no. (2023): 106563.<http://dx.doi.org/10.1016/j.ultsonch.2023.106563>.

[2]	T. Tuziuti,K. Yasui,M. Sivakumar, et al., "Correlation between acoustic cavitation noise and yield enhancement of sonochemical reaction by particle addition," *J. Phys. Chem. A*. 109, no. 21 (2005): 4869-4872.<http://dx.doi.org/10.1021/jp0503516>.

[3]	Y. Gu,B. Li,M. Chen, "An experimental study on the cavitation of water with effects of SiO2 nanoparticles," *Exp. Therm. Fluid Sci.* 79, no. (2016): 195-201.<http://dx.doi.org/10.1016/j.expthermflusci.2016.07.009>.

[4]	A. Shanei,M.M. Shanei, "Effect of gold nanoparticle size on acoustic cavitation using chemical dosimetry method," *Ultrason. Sonochem.* 34, no. (2017): 45-50.<http://dx.doi.org/10.1016/j.ultsonch.2016.05.010>.

[5]	Y. Xie,S. Ren,Y. Gao, et al., "Measuring bulk and surface acoustic modes in diamond by angle-resolved Brillouin spectroscopy," *Science China-Physics Mechanics & Astronomy*. 64, no. 8 (2021): 287311.<http://dx.doi.org/10.1007/s11433-020-1710-6>.

[6]	A.G. Athanassiadis,Z. Ma,N. Moreno-Gomez, et al., "Ultrasound-Responsive Systems as Components for Smart Materials," *Chemical reviews*. 122, no. 5 (2022): 5165-5208.<http://dx.doi.org/10.1021/acs.chemrev.1c00622>.

[7]	B. Cai,J. Mazahreh,Q. Ma, et al., "Ultrasound-assisted fabrication of biopolymer materials: A review," *Int. J. Biol. Macromol.* 209, no. (2022): 1613-1628.<http://dx.doi.org/10.1016/j.ijbiomac.2022.04.055>.

[8]	T.G. McKenzie,F. Karimi,M. Ashokkumar, et al., "Ultrasound and Sonochemistry for Radical Polymerization: Sound Synthesis," *Chem. Eur. J.* 25, no. 21 (2019): 5372-5388.<http://dx.doi.org/10.1002/chem.201803771>.

[9]	K. Melde,E. Choi,Z. Wu, et al., "Acoustic Fabrication via the Assembly and Fusion of Particles," *‌Adv. Mater.* 30, no. 3 (2018): 1704507.<http://dx.doi.org/10.1002/adma.201704507>.

[10]	Y. Yang,T. Ma,S. Li, et al., "Self-Navigated 3D Acoustic Tweezers in Complex Media Based on Time Reversal," *Research*. 2021, no. (2021): 1-13.<http://dx.doi.org/10.34133/2021/9781394>.

[11]	S. Sabrina,M. Tasinkevych,S. Ahmed, et al., "Shape-Directed Microspinners Powered by Ultrasound," *ACS Nano*. 12, no. 3 (2018): 2939-2947.<http://dx.doi.org/10.1021/acsnano.8b00525>.

[12]	H. Bruus, "Acoustofluidics 7: The acoustic radiation force on small particles," *Lab Chip*. 12, no. 6 (2012): 1014-1021.<http://dx.doi.org/10.1039/c2lc21068a>.

[13]	F. Rodriguez,S.K. Patel,C. Cohen, "Measuring the modulus of a sphere by squeezing between parallel plates," *Journal of Applied Polymer Science*. 40, no. 1-2 (1990): 285-295.<http://dx.doi.org/https://doi.org/10.1002/app.1990.070400122>.

[14]	G. van Schie,C. Tanner,P. Snoeren, et al., "Correlating locations in ipsilateral breast tomosynthesis views using an analytical hemispherical compression model," *Physics in Medicine and Biology*. 56, no. 15 (2011): 4715-4730.<http://dx.doi.org/10.1088/0031-9155/56/15/006>.

[15]	A.A. Oberai,F. Roknaldin,T.J.R. Hughes, "Computational procedures for determining structural-acoustic response due to hydrodynamic sources," *Comput. Methods Appl. Mech. Eng.* 190, no. 3-4 (2000): 345-361.<http://dx.doi.org/10.1016/S0045-7825(00)00206-1>.
